# Discrete and overlapping functions of peptidoglycan synthases in growth, cell division and virulence of Listeria monocytogenes

**DOI:** 10.1111/mmi.12873

**Published:** 2014-12-19

**Authors:** Jeanine Rismondo, Lars Möller, Christine Aldridge, Joe Gray, Waldemar Vollmer, Sven Halbedel

**Affiliations:** 1 FG11 Division of Enteropathogenic Bacteria and Legionella, Robert Koch InstituteBurgstrasse 37, 38855, Wernigerode, Germany; 2 ZBS 4 – Advanced Light and Electron Microscopy, Robert Koch InstituteNordufer 20, 13353, Berlin, Germany; 3 Institute for Cell and Molecular Biosciences, The Centre for Bacterial Cell Biology, Newcastle UniversityNewcastle upon Tyne, NE2 4AX, UK; 4 Institute for Cell and Molecular Biosciences, Pinnacle Laboratory, Newcastle UniversityNewcastle upon Tyne, NE2 4HH, UK

## Abstract

Upon ingestion of contaminated food, *Listeria monocytogenes* can cause serious infections in humans that are normally treated with β-lactam antibiotics. These target *Listeria's* five high molecular weight penicillin-binding proteins (HMW PBPs), which are required for peptidoglycan biosynthesis. The two bi-functional class A HMW PBPs PBP A1 and PBP A2 have transglycosylase and transpeptidase domains catalyzing glycan chain polymerization and peptide cross-linking, respectively, whereas the three class B HMW PBPs B1, B2 and B3 are monofunctional transpeptidases. The precise roles of these PBPs in the cell cycle are unknown. Here we show that green fluorescent protein (GFP)-PBP fusions localized either at the septum, the lateral wall or both, suggesting distinct and overlapping functions. Genetic data confirmed this view: PBP A1 and PBP A2 could not be inactivated simultaneously, and a conditional double mutant strain is largely inducer dependent. PBP B1 is required for rod-shape and PBP B2 for cross-wall biosynthesis and viability, whereas PBP B3 is dispensable for growth and cell division. PBP B1 depletion dramatically increased β-lactam susceptibilities and stimulated spontaneous autolysis but had no effect on peptidoglycan cross-linkage. Our *in vitro* virulence assays indicated that the complete set of all HMW PBPs is required for maximal virulence.

## Introduction

*Listeria monocytogenes* is an ubiquitously occurring Gram-positive rod belonging to the firmicutes. It is present in the soil, on plant surfaces or decaying plant material, where it lives as a saprophyte (Freitag *et al*., 2009). However, the bacterium is also able to cause infections in humans upon ingestion of contaminated food. Listeriosis involves self-limiting gastrointestinal symptoms in otherwise healthy individuals but can also develop into more systemic conditions, primarily affecting the brain as well as the fetus in pregnant women (Allerberger and Wagner, 2010). In such severe invasive cases, mortality rates of up to 30% have been reported despite antibiotic therapy (Swaminathan and Gerner-Smidt, 2007). *L. monocytogenes* can induce its uptake into the cytosol of nonphagocytic human host cells via a transient passage through a primary vacuole. The bacterium multiplies inside the host cell cytoplasm from where it can even spread into neighboring cells. This mechanism enables the bacterium to breach all main barriers of the human body (Cossart and Toledo-Arana, 2008). *L. monocytogenes* is sufficiently susceptible to a wide range of antibiotics *in vitro*, but its intracellular growth complicates the antimicrobial treatment of listeriosis due to poor accessibility of antibiotics (Hof, 2004). This is why listeriosis generally requires therapy with high doses of β-lactam antibiotics such as ampicillin or amoxicillin. This treatment can be combined with gentamycin in nonpregnancy cases to achieve synergistic killing effects, as the β-lactams and gentamycin alone are bacteriostatic, whereas the combination of both have bactericidal effects at least *in vitro* (Hof^2003^;^2004^; Allerberger and Wagner, 2010). However, the efficacy of a dual gentamicin-ampicillin therapy has been challenged by a recent epidemiological study, which could not confirm any beneficial effects of such a combination used in a retrospective study on more than hundred cases of invasive listeriosis (Munoz *et al*., 2012). As alternative drugs for anti-listerial treatment, meropenem and cotrimoxazole have also been used with some success (Grant *et al*., 2010; Matano *et al*., 2010; Munoz *et al*., 2012).

**Figure 1:**
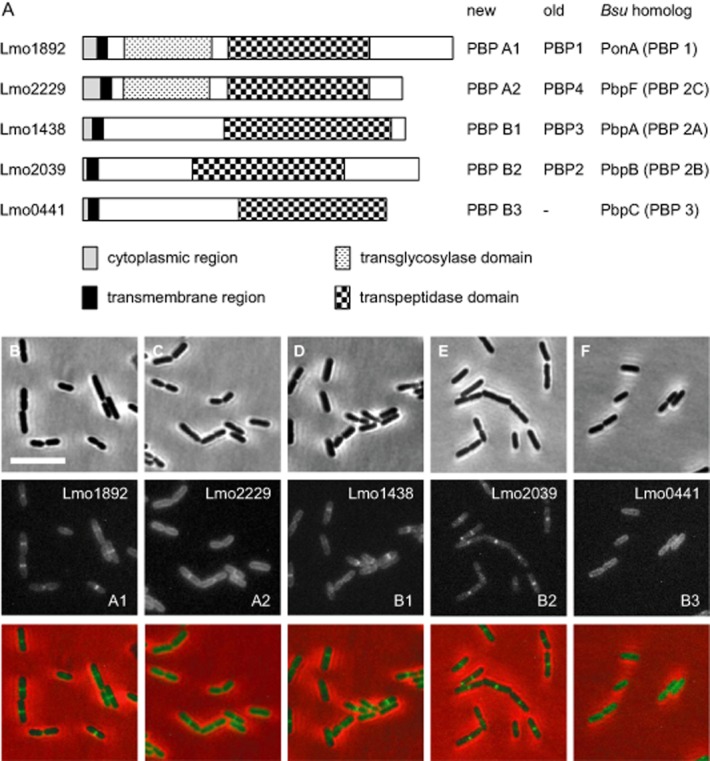
High molecular weight penicillin-binding proteins of *L. monocytogenes* and their subcellular localization.A. HMW PBPs of *L. monocytogenes*. Schematic illustration to show domain organization of the five HMW PBP genes that are present in the *L. monocytogenes* EGD-e genome. PBP names are according to the nomenclature proposed by others (Bierne and Cossart, 2007; Korsak *et al*., 2010). Old PBP names are according to the apparent molecular weights of the individual PBPs on SDS-PAGE gels stained with radiolabeled penicillin (Vicente *et al*., 1990). The corresponding *B. subtilis* homologs are indicated on the right side.B–F. Micrographs showing *L. monocytogenes* cells expressing GFP-PBP fusions. Cells of *L. monocytogenes* LMS44 (*gfp-lmo1892*, B), LMS38 (*gfp-lmo2229*, C), LMS46 (*gfp-lmo1438*, D), LMS45 (*gfp-lmo2039*, E) and LMS47 (*gfp-lmo0441*, F) were grown in BHI broth at 30°C up to mid-logarithmic growth phase and subjected to epifluorescence microscopy (middle panels). Mnemonics in the lower right corners indicate the type of the respective PBPs. Micrographs showing phase contrast images (upper panels) and merged images (lower panels) are included for better comparison. Scale bar is 5 μm.

β-lactam antibiotics covalently bind to and block the active site serine in the transpeptidase domain of penicillin-binding proteins (PBPs) and can therefore be used for their detection using radioactively labeled penicillin. Early studies using this technique uncovered five PBPs in cellular extracts of *L. monocytogenes*, initially denoted PBP1-5 (Vicente *et al*., 1990). Sequence analysis of the *L. monocytogenes* EGD-e genome (Glaser *et al*., 2001) has later confirmed the existence of five high molecular weight (HMW) PBPs and five low molecular weight PBPs (Guinane *et al*., 2006; Bierne and Cossart, 2007; Korsak *et al*., 2010). The five HMW PBPs are also present in the genome of *L. innocua* strain CLIP 11262 and in the 45 *L. monocytogenes* strains for which completely assembled genomes are available at the NCBI server (http://blast.ncbi.nlm.nih.gov, as of 1 August 2014). Class A HMW PBPs are characterized by a short N-terminal cytoplasmic part, a single membrane-spanning region followed by the extracellular transglycosylase and transpeptidase domains. In class B PBPs, the transglycosylase domain is replaced by a noncatalytic domain. *L. monocytogenes* possesses two bi-functional class A HMW PBPs encoded by the *lmo1892* (PBP A1) and the *lmo2229* (PBP A2) genes, and three class B HMW PBPs, encoded by *lmo1438* (PBP B1), *lmo2039* (PBP B2) and *lmo0441* (PBP B3) (Fig. 1A). Deletion and overexpression studies have assigned PBP3 to PBP B1 (Krawczyk-Balska *et al*., 2012), PBP4 to PBP A2 (Zawadzka-Skomial *et al*., 2006; Van de Velde *et al*., 2009) and PBP5 to the D-alanyl-D-alanine-carboxypeptidase PBP D1 (*lmo2754*) (Korsak *et al*., 2005) that cleaves off the terminal D-alanine from murein peptide stems. Inactivation of HMW PBPs of *L. monocytogenes* using gene disruption by integration of temperature sensitive plasmids demonstrated the essentiality of *lmo2039* (encoding PBP B2), whereas the other HMW PBPs were shown to be dispensable for viability suggesting functional redundancies among the HMW PBPs (Guinane *et al*., 2006). Inactivation of PBP A1 or PBP B1 caused severe virulence defects in mice (Guinane *et al*., 2006), and inactivation of PBP A2 and PBP B3 resulted in increased susceptibilities against β-lactams (Guinane *et al*., 2006; Van de Velde *et al*., 2009). These phenotypes suggest that all HMW PBPs contribute to listerial peptidoglycan biosynthesis; however, it is likely that this is achieved via their distinct as well as overlapping physiological functions. Interestingly, PBP A1 genes of *L. monocytogenes* isolates J0161 (reference number: NC_017545.1), J1816 (NC_021829.1) and WSLC1042 (CP007210.1) have acquired premature stop codons leading to truncated proteins lacking up to 110 C-terminal amino acids (Klumpp *et al*., 2014). These truncations do, however, not extend into the transpeptidase domains and, hence, might be without functional consequences. In contrast, more severe truncations are observed in PBP B3 genes of *L. monocytogenes* isolates La111 (NC_020557.1) and N53-1 (NC_020558.1) (Holch *et al*., 2013). In these strains, pre-mature stop codons in the 5′ regions of the PBP B3 genes cause expression of 105 aa long PBP B3 fragments devoid of nearly their complete extracellular domains.

Here we analyzed the contribution of all five HMW PBPs to growth and physiology of *L. monocyctogenes*. GFP fusions of the individual HMW PBPs showed distinct localization patterns suggesting discrete functions. We constructed a set of genetically stable, clean deletion mutants and analyzed their phenotypes. For the essential HMW PBP genes, we generated isopropyl-β-d-thiogalactopyranoside (IPTG)-dependent conditional mutant strains. Our data demonstrated the essentiality of PBP B2 and the synthetic lethality of the two bi-functional PBPs, PBP A1 and PBP A2. The individual HMW PBPs had specific functions in cell growth and/or cell division. Finally, antibiotic susceptibility assays demonstrated that PBP B1 contributes most significantly to the inherent resistance of *L. monocytogenes* against β-lactam antibiotics.

## Results

### Subcellular localization of HMW PBPs

For a better understanding of the role of each listerial HMW PBP, we first determined their subcellular localization. For this purpose, the *gfp* gene was fused to each HMW PBP gene allowing the ectopic expression of GFP-PBP fusion proteins in the wild-type. GFP-PBP A1, GFP-PBP A2 and GFP-PBP B1 were expressed as demonstrated by Western blotting. GFP-PBP B2 and GFP-PBP B3 are expressed at lower levels and showed signs of proteolytic degradation (Fig. S1A). The successful labeling with the fluorescent β-lactam bocillin-fl indicated correct folding of GFP-PBP A1, GFP-PBP A2, GFP-PBP B1 and GFP-PBP B2 (Fig. S1B). Only GFP-PBP B3 was invisible in the bocillin-fl stained gels, even though it was clearly detected in the Western blot. To our knowledge, PBP B3 has never been detected in sodium dodecyl sulfate polyacrylamide gel electrophoresis (SDS-PAGE) gels using labeled penicillins, suggesting that GFP-PBP B3 cannot be stained.

Epifluorescence microscopy showed that GFP-PBP A1 (Fig. 1B), GFP-PBP A2 (Fig. 1C) and GFP-PBP B1 (Fig. 1D) localized to the cell periphery and the septum, albeit to different degrees and not in all cells of the population. In contrast, GFP-PBP B2 localized exclusively to septal sites (Fig. 1E), whereas GFP-PBP B3 produced only peripheral fluorescence signals (Fig. 1F). The distinct subcellular localization patterns possibly reflect functional differences in the listerial HMW PBPs.

### Deletion of *L. monocytogenes* HMW PBP genes

A collection of insertional disruption mutants in HMW PBP genes of *L. monocytogenes*, which had been generated by the help of plasmids containing the temperature sensitive *repA^ts^* allele, was described earlier (Guinane *et al*., 2006). Insertional mutants constructed by such Campbell-like plasmid integrations have, at least in our experience, the tendency to revert in the absence of selection, if the selection pressure for reversion is strong enough. In order to obtain a genetically stable set of mutants in HMW PBP genes of *L. monocytogenes*, we therefore decided to construct strains with marker-less clean deletions of the *lmo1892*, *lmo2229*, *lmo1438*, *lmo2039* and *lmo0441* genes. Derivatives of the pMAD plasmid, allowing scar-less removal of chromosomal sequences (Arnaud *et al*., 2004), were used to generate internal deletions of the *lmo1892* and *lmo2229* genes, encoding the two class A PBPs, PBP A1 and PBP A2 respectively. These deletions were designed in a way that the gene regions comprising the C-terminal part of the transglycosylase domains and N-terminal part of the transpeptidase domains were removed and replaced by premature stop codons (Fig. S2). Likewise, an internal region of *lmo0441*, encoding PBP B3, was removed and replaced by a stop codon (Fig. S2). For removal of the *lmo1438* (PBP B1) and *lmo2039* genes (PBP B2), pMAD derivatives were constructed, which would allow for the removal of the complete open reading frames. However, this strategy was not successful, most likely due to the essentiality of both these genes. In contrast, deletion of both genes was possible in strains LMJR5 and LMJR20, which contained IPTG-inducible, ectopic copies of *lmo2039* and *lmo1438* respectively (Fig. S2). This suggested that PBP A1, PBP A2 and PBP B3 are dispensable, whereas PBP B1 and PBP B2 are essential under standard laboratory conditions.

### Detection of HMW PBPs in *pbp* mutant strains of *L. monocytogenes*

**Figure 2:**
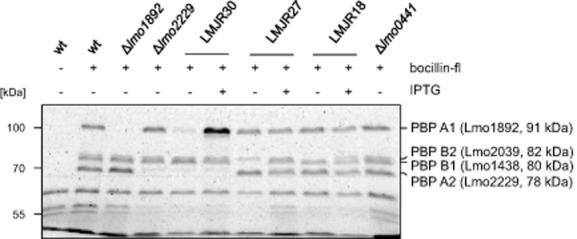
Detection of *L. monocytogenes* penicillin-binding proteins using bocillin-fl. Cells of *L. monocytogenes* EGD-e (wt), LMS57 (Δ*lmo1892*), LMS64 (Δ*lmo2229*), LMJR30 (I*lmo1892* Δ*lmo2229*, I – IPTG-inducible), LMJR27 (I*lmo1438*), LMJR18 (I*lmo2039*) and LMJR41 (Δ*lmo0441*) were grown in BHI broth (containing 1 mM IPTG where indicated) at 37°C to an OD_600_ of 1.0 and membrane fractions were isolated. Aliquots thereof were mixed with bocillin-fl and separated by SDS-PAGE (8% polyacrylamide). Fluorescently labeled PBPs were detected with a Fuji raytest FLA 2000 fluorescence scanner. An unlabeled wild-type control was included to identify nonspecific background fluorescence. Note that the PBP A1/PBP A2 double mutant strain LMJR30 is described in a separate results chapter.

Labeling with the fluorescent penicillin bocillin-fl (Korsak *et al*., 2010) was used to verify the absence or IPTG-dependent expression of HMW PBPs in the constructed mutants. To this end, *L. monocytogenes* strains EGD-e (wild type), LMS57 (Δ*lmo1892*), LMS64 (Δ*lmo2229*), LMJR18 (I*lmo2039*, I – inducible; this designation is used for IPTG-dependent conditional mutants), LMJR27 (I*lmo1438*), as well as LMJR41 (Δ*lmo0441*) were cultivated in brain heart infusion (BHI) broth with or without 1 mM IPTG. Membrane protein fractions were isolated, stained with bocillin-fl and analyzed by 8% SDS-PAGE. The membrane protein extract of the wild-type strain contained four fluorescent protein bands after bocillin-fl staining (Fig. 2). The most prominent band at approximately 100 kDa was absent in extracts from the Δ*lmo1892* mutant LMS57 and therefore corresponds to PBP A1. Extracts of strain LMS64 (Δ*lmo2229*) lacked the second intense band at around 70 kDa, which had previously been assigned to PBP A2 (Zawadzka-Skomial *et al*., 2006; Van de Velde *et al*., 2009). Depletion of PBP B1 (Lmo1438) in strain LMJR27 caused the loss of the lower band of a ∼ 80 kDa-doublet, and depletion of PBP B2 (Lmo2039) in strain LMJR18 led to the loss of the upper band of the same doublet. As indicated in Fig. 2, these results are in good agreement with the theoretical molecular masses of these PBPs. Strain LMJR41 lacking PBP B3 (Lmo0441) showed the same PBP staining pattern as wild type, and the expression of a second ectopic copy of PBP B3 did not produce an additional band (data not shown). PBP B3 bands were also not detected with higher bocillin-fl concentrations (data not shown). This suggests that PBP B3 either generally escapes detection by bocillin-fl or that it is not stable enough to accumulate to a level above the detection limit.

### Growth of *L. monocytogenes* HMW PBP mutants

**Figure 3:**
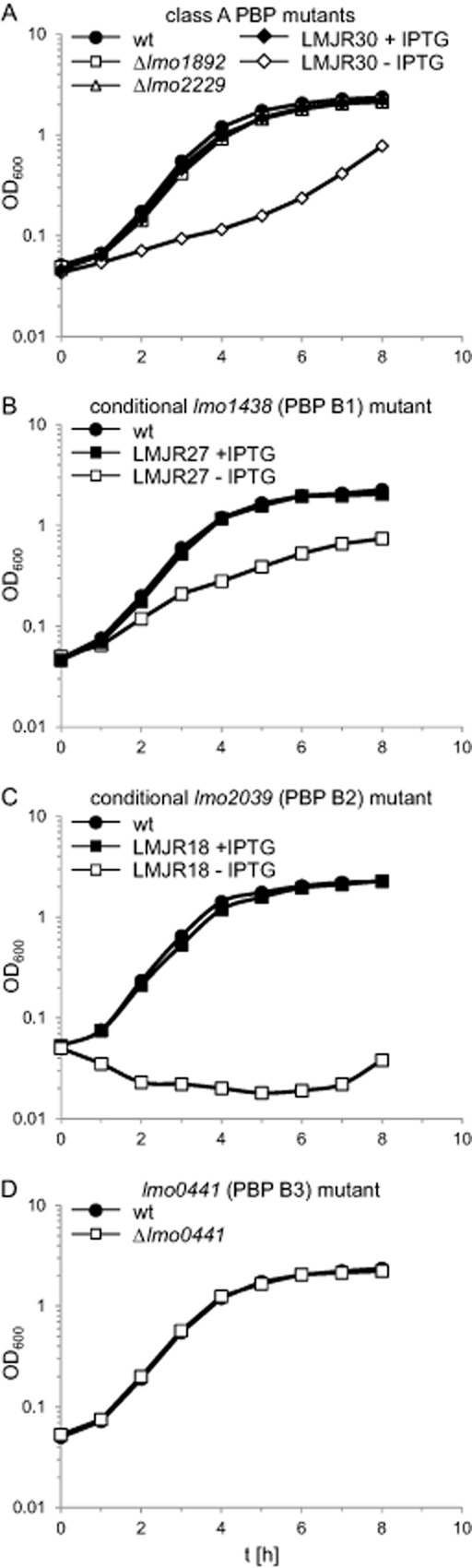
Growth of *L. monocytogenes* strains lacking penicillin-binding proteins.A. Growth of strains lacking class A high molecular weight penicillin-binding proteins PBP A1 (Lmo1892), PBP A2 (Lmo2229) or both. Strains LMS57 (Δ*lmo1892*), LMS64 (Δ*lmo2229*) and LMJR30 (I*lmo1892* Δ*lmo2229*) where cultivated in BHI broth (containing IPTG where indicated) at 37°C and optical density was measured at hourly intervals.B–D. Growth of strains lacking or conditionally expressing class B high molecular weight proteins. Strains LMJR27 (I*lmo1438*, inducible PBP B1), LMJR18 (I*lmo2039*, inducible PBP B2), as well as LMJR41 (Δ*lmo0441*, PBP B3) were cultivated in BHI broth (containing 1 mM IPTG where indicated) and growth was recorded based on optical density.

In order to record possible growth phenotypes, all mutant strains were cultivated in BHI broth and the optical density was measured in hourly intervals. The absence of PBP A1 or PBP A2 did not alter growth under standard laboratory conditions (Fig. 3A) but caused a slight growth defect at 42°C (Fig. S3A). To test the effect of PBP B1 and PBP B2 depletion on growth of *L. monocytogenes*, overnight cultures of strains LMJR27 and LMJR18, respectively, were grown in BHI broth supplemented with 1 mM IPTG and used to inoculate depletion cultures without inducer the next morning. Cells were grown for 24 h at 37°C and used as starting material to inoculate cultures containing or not containing 1 mM IPTG. The depletion of PBP B1 led to a severe reduction of growth of strain LMJR27 (Fig. 3B) at 37°C and a complete growth arrest at 42°C (Fig. S3B). In contrast, the depletion of PBP B2 prevented any growth of strain LMJR18 (Fig. 3C). In order to record the effect of increased temperature on PBP B2 depleted cells, strain LMJR18 was pre-grown in overnight cultures containing IPTG. Cells from these cultures were washed and used to inoculate fresh cultures containing or not containing 1 mM IPTG, and growth was monitored at 37°C and at 42°C. Although there was no growth defect visible at 37°C (Fig. S4A), LMJR18 cells cultivated in the absence of IPTG stopped growth at 42°C, as soon as PBP B2 became depleted, and the cells started to lyse as it is indicated by the constant decline in optical density (Fig. S4B). Finally, strain LMJR41, lacking PBP B3, did not show any growth defects at 37°C or 42°C (Fig. 3D, Fig. S3C). Taken together, these results show that (i) PBP A1, PBP A2 and PBP B3 are not required during growth at 37°C, that (ii) PBP B1 depletion dramatically reduces growth rate, and that (iii) PBP B2 is essential under standard laboratory conditions. Furthermore, loss or depletion of most PBPs causes temperature sensitive phenotypes.

### Cellular morphology of HMW PBP mutants

**Figure 4:**
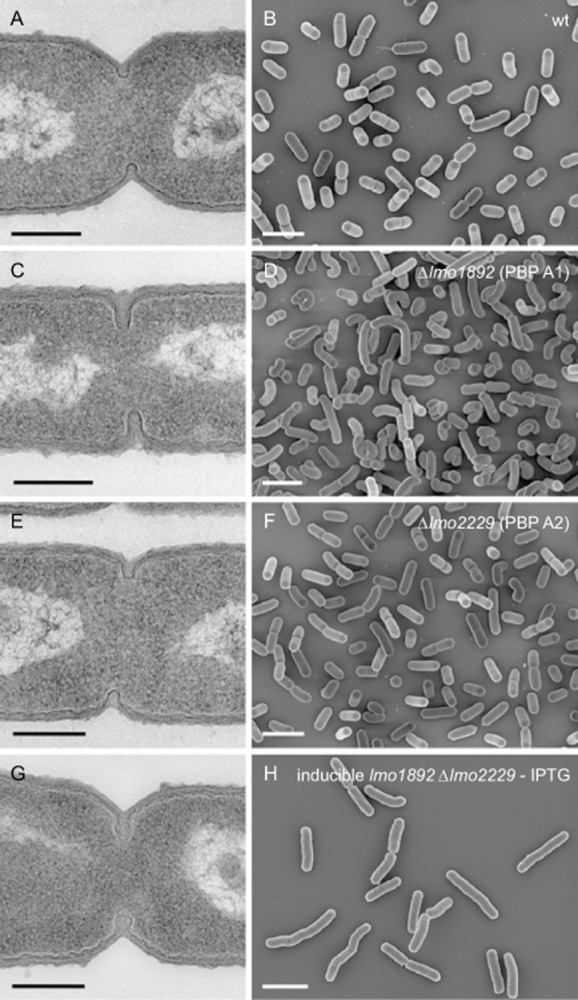
Effect of PBP A1 and PBP A2 inactivation on cell morphology. Transmission electron microscopy of ultrathin sections (A, C, E, G) and scanning electron microscopy (B, D, F, H) of fixed whole cells of *L. monocytogenes* strains devoid of class A high molecular weight penicillin-binding proteins. *L. monocytogenes* strains EGD-e (wt), LMS57 (Δ*lmo1892*), LMS64 (Δ*lmo2229*) and the inducible double mutant strain LMJR30 (I*lmo1892* Δ*lmo2229*) were grown to mid-logarithmic growth phase in BHI at 37°C and subjected to chemical fixation and subsequent electron microscopy as described in the experimental procedures section. Scale bars: left column 200 nm, right column 2 μm.

**Figure 5:**
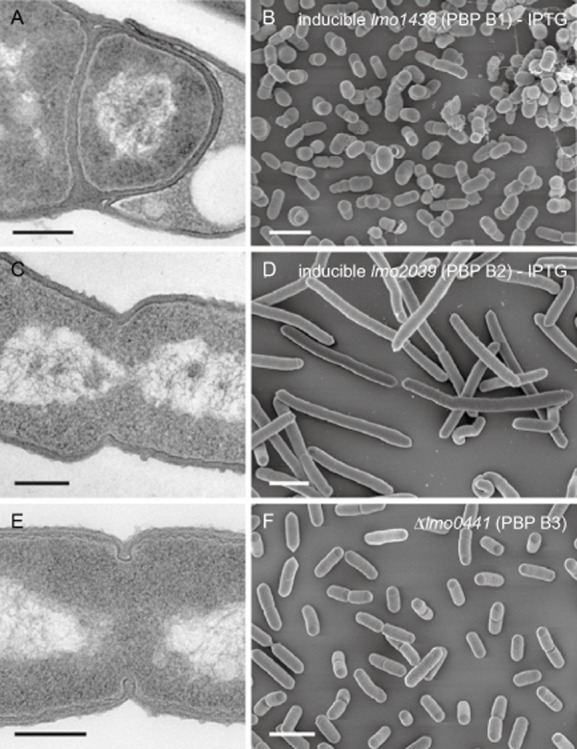
Effect of PBP B1, PBP B2 and PBP B3 inactivation on cell morphology. Transmission electron microscopy of ultrathin sections (A, C, E) and scanning electron microscopy (B, D, F) of fixed whole cells of *L. monocytogenes* strains devoid of class B high molecular weight penicillin-binding proteins. *L. monocytogenes* strains LMJR27 (I*lmo1438*), LMJR18 (I*lmo2039*) and LMJR41 (Δ*lmo0441*) were grown to mid-logarithmic growth phase in BHI at 37°C and subjected to chemical fixation and subsequent electron microscopy as described in the experimental procedures section. Scale bars: left column 200 nm, right column 2 μm.

To better characterize the contribution of the listerial HMW PBPs to cell wall assembly, ultrathin sections of all five *pbp* mutant strains were subjected to transmission electron microscopy. Cells of LMS57 (Δ*lmo1892*, Fig. 4C), LMS64 (Δ*lmo2229*, Fig. 4E) and LMJR41 (Δ*lmo0441*, Fig. 5E) produced normal lateral and septal walls. Septal cross-wall synthesis appeared to be slightly delayed in strain LMS57 (Δ*lmo1892*, Fig. 4C). In sharp contrast, depletion of PBP B1 in strain LMJR27 caused malformed cross-walls, which regularly were found at off-center positions (Fig. 5A). Cells depleted for PBP B1 also seemed to be unable to hydrolyse cross-walls: Often, a thick triangular cell wall (peptidoglycan) mass occupied the transition zones from the lateral walls to the cross-walls; this material is normally degraded with ongoing constriction and cross-wall maturation (Fig. 5A). Depletion of PBP B2 resulted in the initiation of constriction and the formation of small invagination marks at potential division sites, but septation did not continue and cross-walls were not formed (Fig. 5C). Scanning electron microscopy (SEM) showed that lack of PBP A2 and PBP B3 did not cause any changes in cell shape (Figs. 4F and 5F). Lack of PBP A1 in strain LMS57 (Δ*lmo1892*) resulted in slightly elongated and often bent cells (Fig. 4D), indicating a role for PBP A1 in cell division and synthesis of lateral peptidoglycan. Depletion of PBP B1 in cells of strain LMJR27 caused irregularly formed, partially swollen or almost coccoid cells (Fig. 5B), suggesting that PBP B1 primarily functions in lateral peptidoglycan biosynthesis. In good agreement with PBP B2's essential function in cross-wall formation, LMJR18 cells depleted of PBP B2 were extremely elongated (Fig. 5D).

**Figure 6:**
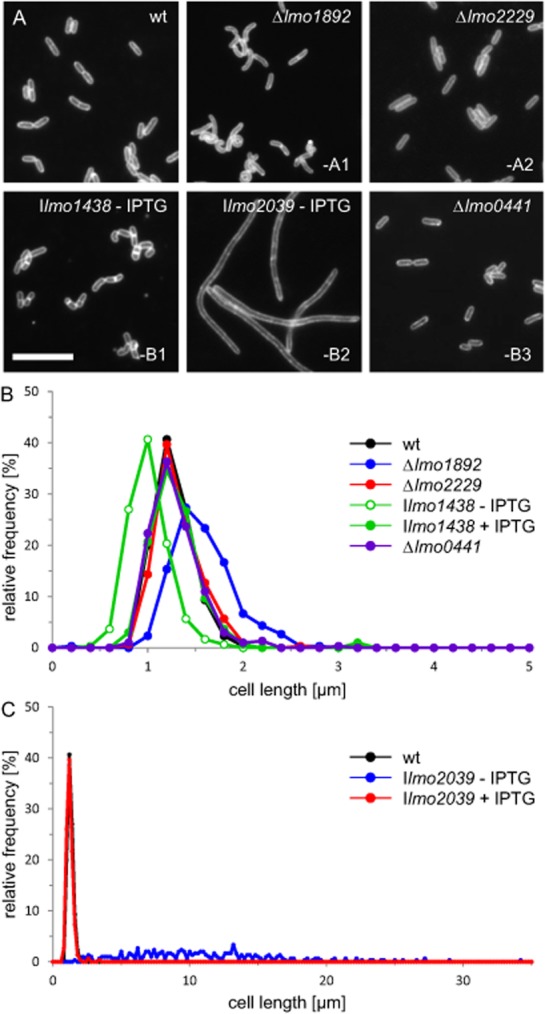
Division phenotypes of *L. monocytogenes pbp* mutants.A. Fluorescence micrographs of *L. monocytogenes pbp* mutant strains after membrane staining using nile red. *L. monocytogenes* strains EGD-e (wt), LMS57 (Δ*lmo1892*), LMS64 (Δ*lmo2229*), LMJR27 (I*lmo1438*), LMJR18 (I*lmo2039*) and LMJR41 (Δ*lmo0441*) were cultivated in BHI broth to mid-logarithmic growth phase. For depletion of PBP B1 (Lmo1438) and PBP B2 (Lmo2039), strains LMJR27 and LMJR18, respectively, were pregrown overnight in the presence of IPTG and then used to start a depletion culture in the absence of IPTG. Mnemonics in the lower right corners indicate the type of the respective deleted or depleted PBPs. (B and C) Cell lengths of 600 cells per strain were measured and visualized as frequency plots. Cell length distribution of PBP B2-depleted cells is illustrated in a separate diagram due to their extreme filamentous growth in the absence of IPTG (C).

For a more precise view on the contribution of the individual HMW PBPs to cell division, we stained all *pbp* mutants with nile red and measured the lengths of 600 cells (Fig. 6A). Consistent with the SEM images, the deletion of PBP A1 caused an increase of the average cell length in strain LMS57 (1.44 ± 0.2 μm) as compared with wild type (1.16 ± 0.2 μm). In contrast, deletion of PBP A2 and PBP B3 in strains LMS64 (1.26 ± 0.2 μm) and LMJR41 (1.18 ± 0.3 μm) did not significantly affect cell length (Fig. 6B). Depletion of PBP B1 led to shorter cells (0.88 ± 0.2 μm, Fig. 6B), whereas PBP B2-depleted cells grew as 10.9 ± 5 μm long, nonseptated filaments (Fig. 6A), whereby the cell lengths were extremely heterogeneous and could reach values of up to 30 μm (Fig. 6C). Taken together, these data demonstrate a general role of PBP A1 in division and maintenance of cell shape. PBP B1 primarily functions in peptidoglycan biosynthesis at the lateral wall, whereas the function of PBP B2 is specific for cross-wall biosynthesis.

### Lack of HMW PBPs weakens the cell wall without changing its composition

The contribution of HMW PBPs to resistance against clinically relevant antibiotics was analyzed by E-tests, using minimum inhibitory concentration (MIC) test strips with antibiotics targeting the cell wall (penicillin, ampicillin, amoxicillin, meropenem and vancomycin) and gentamycin as a control. Susceptibilities to gentamycin and vancomycin did not vary much among the different *pbp* mutant strains. In contrast, deletion or depletion of any of the HMW PBPs caused alterations in the susceptibility to β-lactams (Table 1). Deletion of PBP A1 or PBP A2 resulted in modest effects on β-lactam susceptibility, whereas strong effects were observed with PBP B1 and PBP B2 depleted cells. LMJR18 cells depleted for PBP B2 showed a minor increase in penicillin susceptibility but are two- to threefold more susceptible to meropenem and ampicillin. Depletion of PBP B1 in strain LMJR27 had the strongest effect and caused a three- to fivefold reduction in the MIC for penicillin, ampicillin and meropenem (Table 1). Interestingly, LMJR41 cells lacking PBP B3 also showed an effect as they were roughly twofold more susceptible to penicillin and ampicillin. This was the first indication, which appeared in the course of this study, that the *lmo0441* gene is expressed and that PBP B3 is enzymatically active.

**Figure 7:**
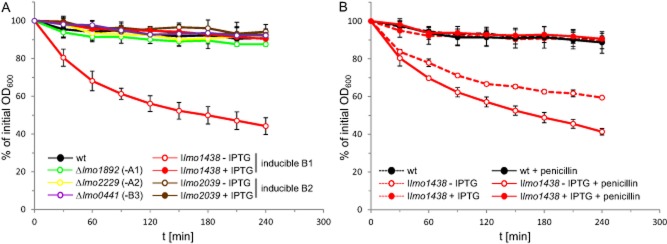
Effect of PBP inactivation on penicillin-induced autolysis.A. Autolysis assay of *L. monocytogenes pbp* mutants. *L. monocytogenes* strains EGD-e (wt), LMS57 (Δ*lmo1892*), LMS64 (Δ*lmo2229*), LMJR27 (I*lmo1438*), LMJR18 (I*lmo2039*) and LMJR41 (Δ*lmo0441*) were pregrown in BHI broth (containing 1 mM IPTG for LMJR18 and LMJR27) at 37°C overnight and used to inoculate fresh BHI medium (containing 1 mM IPTG where indicated). Cultures were grown to mid-log phase (OD_600_∼0.8). Cells were harvested, washed and resuspended in 50 mM Tris-HCl pH 8.0 buffer. Penicillin was added at a concentration of 25 μg ml^−1^, and cells were incubated on a shaker at 37°C.B. Autolysis assay to record the contribution of endogenous autolysis to the effect of PBP B1 depletion on penicillin-induced autolysis. *L. monocytogenes* strains EGD-e (wt) and LMJR27 (I*lmo1438*) were prepared as described in panel A, and the decrease in optical density (λ = 600 nm) over time was recorded photometrically in the presence or absence of penicillin. All values were expressed as relative values. Standard deviations were calculated from experiments performed in triplicate.

Generally, β-lactams do not have a bactericidal effect in *L. monocytogenes*, but PBP inactivation might induce bacteriolysis upon antibiotic treatment. Our quantitative autolysis assays demonstrated that individual deletion or depletion of most HMW PBPs did not induce autolysis in response to penicillin (Fig. 7A). The only exception was the depletion of PBP B1 that drastically enhanced bacteriolysis (I*lmo1438*, Fig. 7A). Roughly two-thirds of this effect was due to spontaneous endogenous autolysis of PBP B1-depleted LMJR27 cells. However, the addition of penicillin further magnifies this effect (Fig. 7B). This result shows that an active PBP B1 is required for cellular integrity and intrinsic penicillin resistance of *L. monocytogenes*.

**Figure 8:**
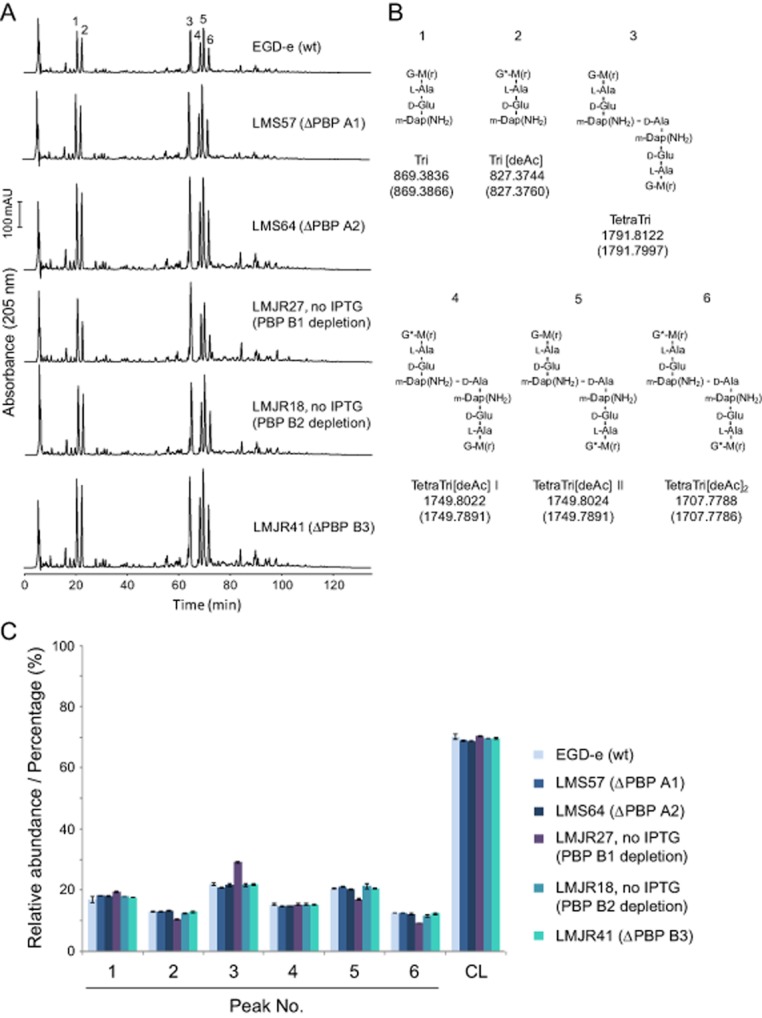
Peptidoglycan composition of *pbp* mutants.A. Muropeptide profiles obtained by HPLC analysis. The strain names and PBP affected are indicated. Major muropeptide peaks are numbered.B. Proposed muropeptide structures corresponding to the major peaks in (A). The names of the muropeptide and the calculated neutral m/z values are given below the structures (upper value, determined neutral m/z; lower value in brackets, theoretical value). G, N-acetylglucosamine; G*, glucosamine; M, N-acetylmuramic acid; *m*-Dap(NH_2_), amidated *meso*-diaminopimelic acid. TetraTri[deAc] exists in two isomers (I and II) in which the positions of the G and G* residues are not known.C. Quantification of the relative abundance of muropeptides 1–6 and of the percent peptides in cross-links (CL) in the peptidoglycan of strains indicated on the right side. The values are the average ± SD of two independent experiments.

We have next analyzed the peptidoglycan composition of PBP-deleted/-depleted cells. The muramidase cellosyl released six major muropeptides that were separated and quantified by high-pressure liquid chromatography (HPLC) (Fig. 8A). Mass spectrometry confirmed the identity of these main fragments (Fig. 8B). Listerial peptidoglycan features an amidated meso-diaminopimelic acid residue at positon 3 of the stem peptide and has a high percentage of glucosamine residues originating from deacetylation of N-acetylglucosamine (Vollmer and Tomasz, 2000; Boneca *et al*., 2007). The different PBP depletion strains showed only minor differences in their muropeptide profile compared with wild type (Fig. 8). The cross-linkage was virtually unaffected, indicating that all combinations of the remaining four HMW PBPs are capable of producing a normally cross-linked peptidoglycan. Cells without PBP B1 had moderately enhanced levels of fully acetylated dimeric muropeptide, indicating a reduced activity of peptidoglycan deacetylase in this strain.

### Lack of both bi-functional HMW PBPs is lethal

The effects of individual PBP A1 and PBP A2 knock-outs on *L. monocytogenes* were rather mild, hence both genes might have overlapping functions and could possibly complement each other. In order to test this hypothesis, the temperature-sensitive plasmid pSH243 was introduced into the PBP A1 deletion strain LMS57 (Δ*lmo1892*). pSH243 was designed for disruption of the *lmo2229* gene encoding PBP A2 in a Campbell-type recombination event (Fig. S5C). The resulting strain (LMS143) could grow in the presence of erythromycin at the permissive temperature (30°C, Fig. S5A), when plasmid replication was unaffected. However, it did not form colonies under restrictive conditions (42°C), due to integration of pSH243 into the chromosome to confer erythromycin resistance. In contrast, strain LMS141 (wild-type background) formed colonies even at 42°C (Fig. S5B) and these colonies had pSH243 integrated in the *lmo2229* open reading frame (data not shown). These results indicated that the simultaneous inactivation of both bi-functional HMW PBPs is not tolerated in *L. monocytogenes*.

In order to obtain a conditional PBP A1 PBP A2 double mutant, an internal deletion of *lmo2229* was generated in strain LMJR21 (Δ*lmo1892* pIMK3*-lmo1892*). The resulting strain (LMJR30) was devoid of PBP A2, whereas the expression of PBP A1 was dependent on IPTG (Fig. 2). Growth of LMJR30 cells was retarded when no IPTG was added to the culture as compared with the presence of the inducer and dramatically lagged behind that of PBP A1 and PBP A2 single mutants (Fig. 3A). Background expression of PBP A1 (Fig. 2) presumably accounted for residual growth in the absence of IPTG. The growth defect of strain LMJR30 was even more pronounced at 42°C, where cells went through only one to two doublings during the 8 h interval, as long as no IPTG was present (Fig. S3A). Obvious ultrastructural changes other than that seen for the PBP A1 mutant LMS57 were not observed for the inducible double mutant (Fig. 4G). However, the slightly increased susceptibilities of the PBP A1 and PBP A2 single mutants to various β-lactam antibiotics appeared to be additive, resulting in a stronger reduction of resistance against penicillin, ampicillin, amoxicillin and meropenem in the conditional double mutant strain, whereas its susceptibilities to vancomycin and gentamycin were unaffected (Table 1). Taken together, depletion of PBP A1 in a ΔPBP A2 background severely impairs growth, heat sensitivity as well as resistance against β-lactams.

### Infection experiments

**Figure 9:**
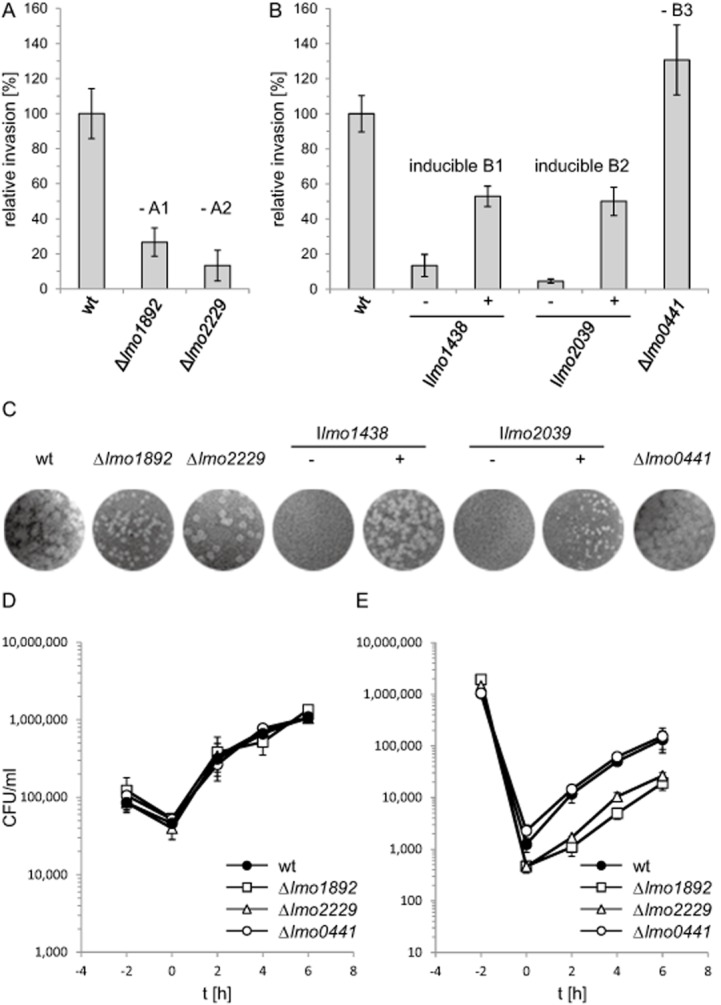
Attenuation of *L. monocytogenes pbp* mutants in *in vitro* infection models.A and B. Invasion of *L. monocytogenes* into HeLa cells. (A) *L. monocytogenes* strains lacking class A HMW PBPs, LMS57 (Δ*lmo1892*) and LMS64 (Δ*lmo2229*) were grown in BHI broth and used to infect HeLa cells. Noninvasive bacteria were washed away and killed using gentamycin. Invasion rates are the ratios of the number of intracellular bacteria versus the number of initially applied bacteria in the inoculum. Values are expressed relative to wild type levels (*n* = 3). (B) *L. monocytogenes* strains conditionally expressing or lacking class B HMW PBPs, LMJR18 (I*lmo2039*), LMJR27 (I*lmo1438*) and LMJR41 (Δ*lmo0441*) were tested in the same way as described for panel A in a separate experiment. Strains LMJR18 and LMJR27 were cultivated in BHI broth either containing (+) or not containing IPTG (−). Bacterial samples recovered from the HeLa cells were plated on BHI agar plates containing 1 mM IPTG for CFU counting.C. Plaque formation assays of *L. monocytogenes pbp* mutant strains. Same set of strains as in panels A and B was used to infect 3T3 mouse embryo fibroblasts. IPTG-dependent strains (LMJR18 and LMJR27) were cultivated in the presence of IPTG prior to infection to ensure wild type-like invasion, but after infection the infected cell cultures were either incubated in the absence or presence of 1 mM IPTG. Zones of killed host cells resulted from spreading bacteria and appeared after 3 days post infection and counterstaining with neutral red.D. Intracellular replication of *L. monocytogenes* PBP A1, PBP A2 and PBP B3 deletion mutants in mouse macrophages. *L. monocytogenes* strains EGD-e (wt), LMS57 (Δ*lmo1892*), LMS64 (Δ*lmo2229*) and LMJR41 (Δ*lmo0441*) were grown in BHI broth at 37°C and used to infect J774.A1 mouse ascites macrophages at a multiplicity of infection of 0.167. Sampling was performed right after infection (*t* = 0 h) and then in intervals of 2 h.E. Intracellular multiplication of the same set of strains in HeLa cells. Experiments were performed as described in the experimental procedures section. Average values and standard deviations were calculated from experiments performed in triplicate.

In order to test whether inactivation of HMW PBPs would impair pathogenicity of *L. monocytogenes*, several *in vitro* infection assays were performed. First, the ability of the HMW PBP mutants to invade nonphagocytic eukaryotic host cells was analyzed in invasion experiments using HeLa cells. In our experimental setup, 1.5 ± 0.2% (*n* = 3) of the wild type cells from the initially applied inoculum invaded the HeLa cells, which is comparable with reported invasion rates (Gaillard *et al*., 1991). In contrast, invasion rates of the strains lacking PBP A1 and PBP A2 were reduced approximately four- and sixfold, respectively (Fig. 9A), indicating a special role for class A HMW PBPs during entry into eukaryotic cells. In order to measure the effect of PBP B1 and PBP B2 depletion, strains LMJR27 and LMJR18 were grown over night in BHI broth containing 1 mM IPTG and were used to inoculate fresh cultures with or without 1 mM IPTG the next morning. When the cultures had reached an OD_600_ of 0.4, the bacteria were harvested, processed and used for infection. Depletion of PBP B1 resulted in a roughly sevenfold reduction of invasion rate in strain LMJR27 when compared with the wild-type situation (13.4 ± 6.3% of wild-type level). Under conditions of PBP B1 expression, however, the invasion rate of LMJR27 cells reached 52.9 ± 5.8% of the wild-type level (Fig. 9B). Likewise, PBP B2 depletion in strain LMJR18 led to a 22-fold reduction of the invasion rate (4.5 ± 1.2% of wild-type level), whereas an invasion efficiency corresponding to 50.1 ± 8.1% of wild type level was observed for LMJR18 cells that had been cultivated in the presence of IPTG (Fig. 9B). In contrast, invasion rate of the PBP B3 deletion mutant LMJR41 slightly exceeded that of the wild type strain (130.1 ± 19.9% of the wild type) (Fig. 9B).

The capability of the HMW PBP mutants to spread from cell to cell was analyzed in a plaque formation assay using 3T3 mouse embryo fibroblasts. *L. monocytogenes* cells infect this cell type and spread circularly from the initial point of infection, forming zones of killed host cells that appear as bright plaques after neutral red staining (Fig. 9C). In the absence of PBP A1, the size but not the number of these plaques was considerably reduced. In contrast, absence of PBP B2 primarily reduced the plaque number but had no effect on the plaque size. This indicates that PBP A1 is required for cell-to-cell spread but does not affect the invasiveness, whereas deletion of PBP A2 has the opposite effect. The contribution of PBP B1 and PBP B2 to cell-to-cell spread was assessed by using LMJR27 and LMJR18 bacteria, which were precultivated in the presence of IPTG to allow wild type–like host cell invasion. After invasion, the infected cell cultures were either left without IPTG or cultivated in the presence of IPTG. In the absence of IPTG, both strains were unable to form plaques, whereas plaques were observed in the presence of the inducer, albeit with reduced size. In contrast, absence of PBP B3 did not affect plaque formation at all. These results demonstrate that PBP A1, PBP B1 and PBP B2 play crucial roles for cell-to-cell spread of *L. monocytogenes*, whereas PBP A2 and PBP B3 are of minor importance for spreading.

The multiplication of mutants lacking PBP A1, PBP A2 or PBP B3 inside eukaryotic cells was investigated using the J774 mouse ascites macrophage cell line. All strains were phagocytosed at a similar rate and grew like the wild-type strain inside these cells (Fig. 9D). When the same set of strain was used to infect HeLa cells, strains lacking PBP A1 and PBP A2 again showed the above-mentioned invasion defect, but all strains multiplied inside HeLa cells with a rate comparable with that of the wild-type strain EGD-e (Fig. 9E).

### Cell wall retention of surface proteins

The cell wall is an anchor for surface proteins that covalently or noncovalently interact with the peptidoglycan (via LPXTG motifs or LysM domains, respectively) or with lipoteichoic acids (via GW modules) (Bierne and Cossart, 2007). In order to test, whether the surface attachment of such proteins is affected in the *pbp* mutant strains, we analyzed cell wall retention of all three protein classes by Western blotting. Internalin A (InlA) is a LPXTG protein (Lebrun *et al*., 1996). It was present in preparations of cell wall associated proteins of all *pbp* mutants and even slightly enriched upon depletion of PBP B1 and PBP B2 (Fig. S6A). Only background InlA levels were observed in culture supernatants of the *pbp* mutants indicating efficient cell wall retention. In contrast, InlA is released from the peptidoglycan and secreted into the growth medium in a Δ*srtA* mutant (Fig. S6A). This strain lacks sortase A required for linking LPXTG proteins to the peptidoglycan (Bierne *et al*., 2002). Likewise, the internalin-like LPXTG protein Lmo0610 (Bierne and Cossart, 2007) is present in cell wall fractions of all *pbp* mutants, and its cell wall retention is lost in a Δ*srtA* mutant (Fig. S6B). Lmo0880 is another LPXTG protein with a yet unknown function (Quereda *et al*., 2013) and also contains a LysM domain (Bierne and Cossart, 2007). Its cell wall retention was not affected in any of the pbp mutants, but SrtA-dependent like the other LPXTG proteins (Fig. S6C). Remarkably, the amount of secreted Lmo0880 was reduced in PBP B1 depleted cells (Fig. S6C). As with the LPXTG proteins, no clear differences in surface retention of the autolysins p60 and MurA (both containing LysM domains) or internalin B (GW protein) were observed in strains lacking PBP A1, PBP A2, PBP B2 or PBP B3 and, as expected, deletion of *srtA* had also no effect (Fig. S6D–F). Cells depleted for PBP B1 had reduced extracellular levels of p60 and MurA, which is consistent with the observed defects in cross-wall degradation (Fig. 5A). Reduction of extracellular p60, MurA and Lmo0880 levels might be explained by the massive lysis of PBP B1-depleted cells, as a result of which extracellular proteins could possibly be degraded by cytosolic proteases.

## Discussion

The Achilles' heel of *L. monocytogenes* having the strongest clinical relevance for antibiotic therapy is the family of PBPs. More than 20 years ago, Vicente *et al*. suggested that the primary lethal target of β-lactam antibiotics of *L. monocytogenes* must be PBP3 (Vicente *et al*., 1990), which we here confirm to be PBP B1 by inducible expression of *lmo1438* and subsequent bocillin-fl staining of membrane protein extracts. Depletion of PBP B1 had indeed the strongest effect on antibiotic susceptibility of *L. monocytogenes* dramatically reducing the MICs to all β-lactams tested. Similar effects on antibiotic resistance were not observed when any of the other HMW PBPs were inactivated. Depletion of PBP B1 furthermore predisposes *L. monocytogenes* to autolysis, and this effect could be further enhanced by addition of penicillin, whereas inactivation of any of the other HMW PBPs did neither stimulate spontaneous nor penicillin-induced autolysis. These results suggest that the other two transpeptidases, PBP B2 and PBP B3, must have rather specific (PBP B2) or only secondary (PBP B3, see below) functions in peptidoglycan cross linking, which are less important for maintenance of general cellular integrity. However, our data also show that transpeptidation of peptidoglycan strands by PBP B1 is required for the intrinsic resistance of *L. monocytogenes* against β-lactam antibiotics and thus supports the initial suggestion by Vicente (Vicente *et al*., 1990). Analysis of muropeptide composition showed normal peptidoglycan cross-linkage upon PBP B1 depletion but reduced levels of GlcNAc deacetylation. The latter could enhance the activity of endogenous peptidoglycan hydrolases like the muramidase MurA (NamA) or the glucosaminidase Auto (Carroll *et al*., 2003; Cabanes *et al*., 2004; Bublitz *et al*., 2009), leading to increased autolysis. That individual inactivation of none of the other PBPs had an effect on the muropeptide pattern is likely explained by the redundancy of the PBPs, which may compensate for each other.

A major problem in listeriosis treatment using β-lactams is that these antibiotics do not induce efficient bacteriolysis (Hof, 2004; Lemaire *et al*., 2005; Grayo *et al*., 2008). Remarkably, the β-lactams with the lowest MICs against *L. monocytogenes*, are the ones that exhibit the highest binding affinities to PBP B1, and these are penicillin G and imipenem (Vicente *et al*., 1990). This shows that anti-listerial intervention strategies with these antibiotics already target the PBP whose inactivation causes the strongest bacteriolytic effect. However, inactivation of PBP B1 by β-lactams can only prevent transpeptidation from the time of treatment onward. This is the difference to our depletion experiments and therefore resolves the obvious contradiction between the low bacteriolytic activities of β-lactams (Lemaire *et al*., 2005) and the strong induction of autolysis upon PBP B1 depletion. Compounds inhibiting PBP B1 by a different molecular mechanism or with a higher efficiency than the β-lactams could possibly improve antibiotic therapy of listeriosis by promoting bacteriolysis *in situ*.

In contrast to the pleiotropic PBP B1 phenotype, inactivation of PBP B2 activity through depletion of the *lmo2039* gene product led to a more specific defect and was limited to septum formation and cross-wall biosynthesis. PBP B2 is homologous to PBP 2B (PbpB) of *Bacillus subtilis*, which has been described as one of the late division proteins that is recruited to the divisome after assembly of the FtsZ-ring (Daniel *et al*., 2000; Gamba *et al*., 2009). Depletion of *B. subtilis* PBP 2B causes a block in cell division leading to filament formation as is observed here with PBP B2-depleted *L. monocytogenes* cells. FtsZ-rings still assemble in PBP 2B-depleted cells of *B. subtilis*, but septal recruitment of late division proteins, such as DivIC and DivIB, is severely impaired, preventing completion of divisome assembly, constriction, cross-wall biosynthesis and cell division (Daniel *et al*., 2000). The function of *L. monocytogenes* PBP B2 seems to be identical to that of *B. subtilis* PBP 2B, and the concept of a divisome-specific role of PBP B2 is in good agreement with the observation that GFP-PBP B2 localized exclusively to the division septa (Fig. 1E). In contrast, inactivation of PBP B3 had no effect on growth, morphology or virulence, consistent with the finding that the lack of its homologue PbpC in *B. subtilis* causes no phenotypical effect (Murray *et al*., 1996). The only phenotype associated with lack of *L. monocytogenes* PBP B3 was a slight reduction in the susceptibility to several β-lactams and a slightly increased invasion efficiency (Table 1, Fig. 9B). This showed that PBP B3 is not a silent gene but rather must be an actively expressed gene. Possibly, its function is redundant and taken over by one of the other class B HMW PBPs in cells lacking PBP B3. It would be interesting to see, if deletion of PBP B3 in the inducible PBP B1 mutant would generate a strain that would not grow in the absence of IPTG at all.

**Table 1:** Antibiotic susceptibilities of *L. monocytogenes* penicillin-binding protein mutants

Strain	Genotype	affected PBP	Minimal Inhibitory Concentration (μg ml^−1^)^a^					
			Penicillin	Ampicillin	Amoxicillin	Meropenem	Vancomycin	Gentamycin
EGD-e	wt	–	0.074 ± 0.017	0.053 ± 0.018	0.0183 ± 0.004	0.048 ± 0.016	1.5 ± 0.5	0.25 ± 0.00
LMS57	Δ*lmo1892*	A1	0.029 ± 0.005	0.053 ± 0.018	0.016 ± 0.00	0.052 ± 0.01	1.33 ± 0.288	0.253 ± 0.109
LMS64	Δ*lmo2229*	A2	0.029 ± 0.005	0.037 ± 0.008	< 0.016	0.034 ± 0.012	1.5 ± 0.05	0.21 ± 0.035
LMJR30 – IPTG	I*1892* Δ*2229*	A1, A2	0.016 ± 0.00	0.016 ± 0.00	< 0.016	0.026 ± 0.005	1.67 ± 0.29	0.25 ± 0.11
LMJR27 – IPTG	I*lmo1438*	B1	< 0.016	< 0.016	< 0.016	0.011 ± 0.004	1.5 ± 0.00	0.31 ± 0.16
LMJR18 – IPTG^b^	I*lmo2039*	B2	0.053 ± 0.01	0.016 ± 0.00	< 0.016	0.023 ± 0.00	1.83 ± 0.29	0.21 ± 0.035
LMJR41	Δ*lmo0441*	B3	0.027 ± 0.009	0.027 ± 0.009	< 0.016	0.042 ± 0.009	1.83 ± 0.29	0.27 ± 0.1

a Antibiotic susceptibilities are expressed as average values of minimal inhibitory concentrations ± standard deviations, calculated from three independent repetitions.

b BHI plates containing 0.025 mM IPTG were used to deplete PBP B2 (Lmo2039).

A synthetic lethal effect was observed with the two bi-functional class A HMW PBPs, PBP A1 and PBP A2, demonstrating that the presence of one of the two is essential for viability of *L. monocytogenes*. There are no other known transglycosylase genes present in the listerial genome, so the inactivation of PBP A1 and PBP A2 genes is expected to halt peptidoglycan biosynthesis. Depletion of PBP A1 in a ΔPBP A2 background resulted in a significant reduction of growth rate. Depleted cells still had background levels of PBP A1 (Fig. 2), explaining why growth was not completely abolished (Fig. 3A). Remarkably, the situation is different in *B. subtilis*: In this species the deletion of all four bi-functional HMW PBPs was tolerated and generated a viable strain, which still synthesized peptidoglycan presumably by an unknown transglycosylase (Popham and Setlow, 1996; McPherson and Popham, 2003). Our results suggest that this activity is not present or is present and cannot support growth of *L. monocytogenes* lacking PBP A2 and PBP A1. Perhaps a simultaneous deletion of both corresponding genes is possible under osmoprotective conditions, which would support stabilization of protoplasted cells, or in the cell wall deficient L-form mode of growth (Dell'Era *et al*., 2009).

Many surface proteins are covalently linked to the cell wall (LPXTG proteins) or associated to peptidoglycan components via noncovalent interactions (proteins containing GW modules or LysM domains). Among the LPXTG proteins are several internalins, including the major internalin InlA. The second major internalin InlB is associated with the cell wall via GW modules, and LysM domains for peptidoglycan binding are present in the two major autolysins p60 and MurA (Machata *et al*., 2005; Bierne and Cossart, 2007). All of these proteins are required for invasion into different cell types (Gaillard *et al*., 1991; Bergmann *et al*., 2002; Pilgrim *et al*., 2003; Halbedel *et al*., 2012), whereas p60 also contributes to cell-to-cell spread (Pilgrim *et al*., 2003). We only observe slight aberrations in cell wall retention of these proteins in some of the *pbp* mutants and the observed changes do not fit with their attenuation patterns in our virulence assays. Hence, distortions in net cell wall retention of any of these proteins do not account for the observed virulence defects. We cannot rule out that the distribution patterns of these proteins on the cell surface are altered or that cell wall retention of other surface proteins, critical for invasion and cell-to-cell spread is affected in the *pbp* mutants. Alternatively, changes in architecture, ultrastructure or physico-chemical properties of the peptidoglycan sacculus *per se* could contribute to the observed effects. Future experiments will have to clarify this question.

## Experimental procedures

### Bacterial strains and growth conditions

All bacterial strains used in this study are listed in Table 2. Cells of *L. monocytogenes* were routinely grown in BHI broth or on BHI agar plates at 37°C if not stated otherwise. If required, antibiotics and supplements were added to the growth media at the following concentrations: erythromycin (5 μg ml^−1^), kanamycin (50 μg ml^−1^), X-Gal (100 μg ml^−1^) and IPTG (1 mM). *Escherichia coli* TOP10 was used as the standard host for all cloning procedures (Sambrook *et al*., 1989).

**Table 2:** Strains and plasmids used in this study

Name	Relevant characteristics	Source/reference
Plasmids		
pAUL-A	*lacZα erm*	(Chakraborty *et al*., 1992)
pIMK3	P*_help_-lacO lacI neo*	(Monk *et al*., 2008)
pMAD	*bla erm bgaB*	(Arnaud *et al*., 2004)
pSH195	P*_help_-gfp neo*	(Halbedel *et al*., 2012)
pUC19	*bla lacZα*	Invitrogen
pJR4	*bla* Δ*lmo1438*	This work
pJR5	*bla erm bgaB* Δ*lmo1438*	This work
pJR7	*bla* Δ*lmo2039*	This work
pJR8	*bla erm bgaB* Δ*lmo2039*	This work
pJR10	P*_help_-lacO-lmo2039 lacI neo*	This work
pJR17	P*_help_-lacO-lmo1438 lacI neo*	This work
pJR18	P*_help_-lacO-lmo1892 lacI neo*	This work
pJR20	*bla erm bgaB* Δ*lmo2229*	This work
pJR24	*bla erm bgaB* Δ*lmo0441*	This work
pSH239	P*_help_-gfp-lmo2229 neo*	This work
pSH243	*erm ‘lmo2229’*	This work
pSH248	P*_help_-gfp-lmo1892 neo*	This work
pSH249	P*_help_-gfp-lmo2039 neo*	This work
pSH250	P*_help_-gfp-lmo1438 neo*	This work
pSH251	P*_help_-gfp-lmo0441 neo*	This work
pSH253	*bla erm bgaB 'gfp-lmo1892*	This work
pSH257	*bla erm bgaB* Δ*lmo1892*	This work
pSH258	P*_help_-gfp*-Δ*lmo2229 neo*	This work
pSH277	P*_help_-gfp*-Δ*lmo0441 neo*	This work
pSH286	P*_help_-lacO-gfp-lmo2039 lacI neo*	This work
*L. monocytogenes* strain**s**		
EGD-e	Wild type, serovar 1/2a strain	Lab collection
BUG1777	Δ*srtA*	(Bierne *et al*., 2002)
LMS22	*erm*	(Halbedel *et al*., 2012)
LMS38	*attB::*P*_help_-gfp-lmo2229 neo*	pSH239 → EGD-e
LMS44	*attB::*P*_help_-gfp-lmo1892 neo*	pSH248 → EGD-e
LMS45	*attB::*P*_help_-gfp-lmo2039 neo*	pSH249 → EGD-e
LMS46	*attB::*P*_help_-gfp-lmo1438 neo*	pSH250 → EGD-e
LMS47	*attB::*P*_help_-gfp-lmo0441 neo*	pSH251 → EGD-e
LMS57	Δ*lmo1892*	pSH257 ↔ EGD-e
LMS64	Δ*lmo2229*	pJR20 ↔ EGD-e
LMS141	*'lmo2229' erm*	pSH243 → EGD-e
LMS142	Δ*lmo1892 erm*	pAUL-A → LMS57
LMS143	Δ*lmo1892 'lmo2229' erm*	pSH243 → LMS57
LMJR5	*attB::*P*_help_-lacO-lmo2039 lacI neo*	pJR10 → EGD-e
LMJR18	Δ*lmo2039 attB::*P*_help_-lacO-lmo2039 lacI neo*	pJR8 ↔ LMJR5
LMJR20	*attB::*P*_help_-lacO-lmo1438 lacI neo*	pJR17 → EGD-e
LMJR21	Δ*lmo1892 attB::*P*_help_-lacO-lmo1892 lacI neo*	pJR18 → LMS57
LMJR27	Δ*lmo1438 attB::*P*_help_-lacO-lmo1438 lacI neo*	pJR5 ↔ LMJR20
LMJR30	Δ*lmo1892* Δ*lmo2229 attB::*P*_help_-lacO-lmo1892 lacI neo*	pJR20 ↔ LMJR21
LMJR41	Δ*lmo0441*	pJR24 ↔ EGD-e

The arrow (→) stands for a transformation event, and the double arrow (↔) indicates gene deletions obtained by chromosomal insertion and subsequent excision of pMAD plasmid derivatives (see *Experimental procedures* for details).

### General methods, manipulation of DNA and oligonucleotide primers

Standard protocols were used for transformation of *E. coli* and isolation of plasmid DNA (Sambrook *et al*., 1989). Generation of electro-competent *L. monocytogenes* cells and transformation of plasmid DNA into *L. monocytogenes* were performed as described (Monk *et al*., 2008). Enzymatic modification of plasmid DNA was carried out as described by the instructions given by the manufacturers. Quickchange mutagenesis was employed for restriction free modification of plasmids (Zheng *et al*., 2004). DNA sequences of oligonucleotide primers are listed in Table 3.

**Table 3:** Oligonucleotides used in this study

Name	Sequence (5′→3′)
SHW164	CTTAGGTACCTTTGTATAGTTCATCCATGCC
SHW165	GACCGCTCGAGCCCAGCTTTTGTTCCCTTTAG
SHW166	CTTAGGTACCGACAAATTCAAACAGCAACTTATTA
SHW167	GACCGCTCGAGTTAATTACCTATCGAATCGATTAAG
SHW182	AATGCAGTAGTTTCCATTGAAG
SHW183	TTATTTGTATTTATCGCCTTCTGC
SHW184	CTTAACTAGTTTTGTATAGTTCATCCATGCC
SHW185	CTTAACTAGTGCAGATAAACCGCAGACAAG
SHW186	GACCGCTCGAGTTAACTATCTGGAATTTTAACAGTGG
SHW187	CTTAACTAGTAAACGGCGTATAGGTAACATG
SHW188	GACCGCTCGAGTTAATTGAGCAAATCACCGATAC
SHW189	CTTAACTAGTAAACTAAATTTTAGAAAAAAGAAAAAAG
SHW190	GACCGCTCGAGTTAATTTTCGGTTTGTTCTGATTG
SHW191	CTTAACTAGTGCTAGTTATGGTGGGAAAAAGAG
SHW192	GACCGCTCGAGTTATTTATACATACTTTCAATTACAG
SHW215	CACGCGTCGACTTAGTGAACACGGTCTGACATATAG
SHW216	CACGCGTCGACGGAGCAAACAGTTCCAACCC
SHW217	CACGCGTCGACTTACCACTCCCCATTACCAAAG
SHW218	CACGCGTCGACTCCACGATGAAGCCACTCG
SHW271	AAAGTGCGCATCTAAGGCTTGAAAAAATTTGATTTTGATAAG
SHW272	TTTTTTCAAGCCTTAGATGCGCACTTTATCGGTTTTC
JR10	CGGGATCCATGAGCAGAAAAATTGCAAGCATT
JR11	ATATGTCGACTTTCACGCATAGTTACCTCACTTT
JR12	ATATGTCGACAATTAATCAAAAACCACTTTCATTTAT
JR13	GCATGCCATGGAGTCATTCTCTGTGAAAACGTCAA
JR20	ATACGAGATCTGTCGTCGCGAAGTGAAACCAATTG
JR33	TATTAGTCGACTTTCATTGACCATCTACCACTTTC
JR34	TGAAAGTCGACTAATAATAGGAAGATAGAAGTATGTC
JR35	CGTGGATCCGATCTTGAGACAAATTCATAAAC
JR48	ACGCCGTTTATCCTTTTCCATGGGTTTCAC
JR49	AAGGATAAACGGCGTATAGGTAACATGAG
JR53	GCGCCCATGGCAGATAAACCGCAGACAAG
JR54	GCGCGGATCCTTAACTATCTGGAATTTTAACAGTGG
JR57	TCCCCCCGGGCAAACTAAATTTTAGAAAAAAGAAAAAAGATTC
JR58	GCCCATCGATTTAATTTTCGGTTTGTTCTGATTGTGC

### Construction of strains expressing fluorescent fusion proteins

In order to obtain fluorescent variants of PBP proteins, GFP was fused to the N-terminus of all five HMW PBPs. The *lmo2229* open reading frame was amplified in a polymerase chain reaction (PCR) using the primers SHW166/SHW167 and KpnI/XhoI-cloned into the backbone of plasmid pSH195 that had been linearized by PCR using the oligonucleotides SHW164/SHW165 and cut with the same enzymes. This resulted in plasmid pSH239. Likewise, the *lmo1892* (SHW185/186), *lmo2039* (SHW187/SHW188), *lmo1438* (SHW189/SHW190) and *lmo0441* (SHW191/SHW192) open reading frames were amplified and SpeI/XhoI-cloned into pSH195 which, however, had been linearized in a PCR using the primers SHW184/SHW165 and cut with SpeI/XhoI. The obtained plasmids were sequenced and named pSH248, pSH249, pSH250 and pSH251 respectively.

Plasmids pSH239 and pSH248-pSH251 were introduced into *L. monocytogenes* strains by electroporation, and kanamycin resistant clones were selected. Plasmid insertion at the *attB* site of the tRNA^Arg^ locus was verified by PCR.

### Construction of *pbp* mutant strains

For deletion of an internal fragment of *lmo1892* from the *L. monocytogenes* chromosome, plasmid pSH257 was constructed, which was obtained in two steps. First, the BamHI/XhoI *'gfp-lmo1892* fragment of plasmid pSH248 was cloned into pMAD, which had been linearized with BamHI/SalI. From the resulting plasmid (pSH253), a fragment corresponding to *lmo1892* amino acid codons 206–485 was replaced by a TAA stop codon and a SalI site, using PCR and primers SHW215/SHW216. The resulting fragment was cut with SalI, self-ligated and yielded plasmid pSH257 upon transformation.

Plasmid pJR20 was constructed to facilitate deletion of an internal *lmo2229* fragment. First, an internal fragment corresponding to amino acid residues 205–393 of the *lmo2229* open reading frame of plasmid pSH239 was replaced by a stop codon followed by a SalI site in a PCR using the primer pair SHW217/SHW218. The obtained PCR fragment was cut with SalI, self-ligated and resulted in plasmid pSH258 after transformation. The EcoRI/BamHI *'gfp*-Δ*lmo2229* fragment of plasmid pSH258 was then subcloned into EcoRI/BamHI cut pMAD to give plasmid pJR20. Plasmid pSH243, designed for insertional disruption of *lmo2229*, was constructed by blunt end cloning of an internal *lmo2229* fragment, which was obtained using primers SHW182/SHW183, into SmaI cut pAUL-A. The reverse primer, SHW183, introduced a premature stop codon.

For deletion of the *lmo1438* gene, plasmid pJR5 was constructed in two steps. First, up- and downstream regions of the *lmo1438* gene were amplified using the primers JR10/JR11 and JR12/JR13 respectively. Both fragments were joined by ligation after their ends had been made compatible by SalI digestion and the joined fragment was amplified from the ligation mixture by PCR using JR10/JR13 as the primers. This fusion fragment was then blunt-end cloned into SmaI cut pUC19 and subcloned from the resulting plasmid (pJR4) into pMAD using NcoI restriction digestion.

Plasmid pJR8 was constructed for the removal of the *lmo2039* gene and was generated in a similar way. Up- and downstream regions of *lmo2039* were PCR amplified using primers JR20/JR33 and JR34/JR35, respectively, mixed and used as a template in a small overlapping extension PCR with the primer pair JR20/JR35. The fusion product was blunt-end cloned into SmaI cut pUC19 and subcloned from the resulting plasmid (pJR7) into pMAD using BamHI/BglII as the restriction enzymes.

To facilitate deletion of *lmo0441* from the chromosome, plasmid pJR24 was generated. To this end, an internal *lmo0441* fragment, corresponding to amino acids 168–501, was replaced by a premature stop codon from plasmid pSH251 in a PCR using the primer pair SHW271/SHW272. The BamHI/XhoI '*gfp*-Δ*lmo0441* fragment of the resulting plasmid (pSH277) was then subcloned into BamHI/EcoRI cut pMAD.

For IPTG-inducible expression of *lmo2039*, the BamHI/XhoI *gfp-lmo2039* fragment of plasmid pSH249 was first subcloned into BamHI/SalI cut pIMK3, resulting in plasmid pSH286. Later, the *gfp* part of the *gfp-lmo2039* fusion of pSH286 was removed in a PCR using the primer pair JR48/JR49, and the resulting plasmid was named pJR10. For inducible expression of *lmo1438*, the *lmo1438* open reading frame was amplified from chromosomal DNA with primers JR57/JR58, cut with ClaI/XmaI and ligated to the ClaI/XmaI cut backbone of pIMK3. This resulted in plasmid pJR17. Plasmid pJR18 was constructed to allow for IPTG-controllable expression of *lmo1892*. It was generated by amplification of the *lmo1892* open reading frame, which had been obtained in a PCR using primers JR53/JR54, into pIMK3 after NcoI/BamHI digestion. Plasmids pJR10, pJR17 and pJR18 were inserted into the *attB* site of the tRNA^Arg^ locus as mentioned above. The chromosomal *pbp* genes were deleted in strain EGD-e or in strains containing ectopic, IPTG-controllable *pbp* genes using plasmids pSH257, pJR5, pJR8, pJR20, pJR24 and a plasmid integration/excision strategy described by others (Arnaud *et al*., 2004).

### Isolation, separation and detection of proteins

Cells were cultivated in BHI broth at 37°C and harvested by centrifugation when the culture reached an optical density of 1.0. The cell pellet was washed once with ZAP buffer (10 mM Tris/HCl pH 7.5 and 200 mM NaCl), resuspended in 1 ml ZAP buffer also containing 1 mM phenylmethylsulfonyl fluoride (PMSF) and disrupted by sonication. Cell debris was removed by centrifugation (1 min, 13.000 rpm in a table top centrifuge), and membrane proteins were collected from the resulting supernatant by ultracentrifugation at 100.000 × *g* for 30 min at 4°C. The membrane fraction in the resulting pellet was resuspended in 100 μl ZAP buffer. In order to detect PBPs in SDS-PAGE gels, aliquots corresponding to 20 μg of membrane proteins were incubated with 3 μM bocillin-fl (Molecular Probes) for 20 min at 37°C. The binding reaction was stopped by addition of loading dye and incubation for 5 min at 65°C. Samples were separated by SDS-PAGE (8% acrylamide, 0.067% bis-acrylamide), and PBPs were detected using a Fuji raytest FLA 2000 fluorescence scanner.

For isolation of surface proteins, a previously published protocol was used with minor modifications (Jonquieres *et al*., 1999). Bacteria were grown in 50 ml BHI broth at 37°C until an OD_600_ of 2.0 and harvested by centrifugation. Proteins from the culture supernatant were precipitated using trichloroacetic acid (TCA) (Halbedel *et al*., 2014), and the cell pellet was washed once with phosphate buffered saline (PBS), once with TS buffer (10 mM Tris-HCl pH 6.9, 10 mM MgCl_2_, 0.5 M sucrose) and resuspended in 1.5 ml TS buffer containing 1 mM PMSF, 100 μg ml^−1^ lysozyme and 250 μg ml^−1^ RNase. After incubation for 1 h at 37°C with gentle shaking, cells were removed by centrifugation (13.000 rpm for 5 min in a table top centrifuge), and the released surface proteins were precipitated from the supernatant by addition of TCA (16% final concentration) over night at 4°C. Precipitated proteins were collected by centrifugation and dissolved in 100 μl 8 M urea.

In order to detect proteins by Western blotting, protein extracts were separated by standard SDS polyacrylamide gel electrophoresis and transferred onto positively charged polyvinylidene fluoride (PVDF) membranes using a semi-dry transfer unit. Proteins were immune-stained using polyclonal rabbit antisera recognizing GFP (lab stock), Lmo610 and Lmo0880 (Quereda *et al*., 2013) or monoclonal mouse antibodies against InlA, InlB (Lingnau *et al*., 1995) and p60 (MyBioSource, USA) as the primary and anti-rabbit or anti-mouse immunoglobulins G conjugated to horseradish peroxidase as the secondary antibody. The ECL(Tm) chemiluminescence detection system (Thermo Scientific) was used for detection of the peroxidase conjugates on the PVDF membrane. Zymography was performed as described earlier (Halbedel *et al*., 2014).

### Determination of minimal inhibitory concentrations

Test strips with the following concentration gradients were used to determine the MICs of selected antibiotics: Amoxicillin (0.016–256 μg ml^−1^), ampicillin (0.016–256 μg ml^−1^), gentamicin (0.016–256 μg ml^−1^), meropenem (0.002–32 μg ml^−1^), penicillin G (0.016–256 μg ml^−1^), vancomycin (0.016–256 μg ml^−1^, all from Bestbiondx, Germany). *L. monocytogenes* strains were cultivated on BHI agar, containing 1 mM IPTG where necessary, at 37°C overnight, and several colonies were resuspended in 5 ml BHI broth. This resuspension was used to swab-inoculate BHI agar plates. MIC test strips were placed on top of the agar surface, and the plates were incubated at 37°C for 1 day.

### Autolysis assay

Bacterial strains were cultivated in BHI broth (containing 1 mM IPTG where required) at 37°C up to an OD_600_ of around 0.8. Cells were collected by centrifugation (6000 × *g*, 5 min) and resuspended in 50 mM Tris/HCl pH 8.0 to an optical density of OD_600_ = 0.6. Penicillin was added to a final concentration of 25 μg ml^−1^, and the cells were shaken at 37°C. Autolysis was recorded by measuring the decline in optical density (λ = 600 nm) over time.

### Peptidoglycan analysis

Cell wall from *L. monocytogenes* was isolated as described for *S. pneumoniae* (Bui *et al*., 2012). Wall teichoic acid was released by incubating the cell wall with 48% hydrofluoric acid at 4°C for 48 h, followed by washing of the resulting peptidoglycan as described (Bui *et al*., 2012). Peptidoglycan (2.5 mg ml^−1^) was incubated with 25 μg ml^−1^ of the muramidase cellosyl in 20 mM sodium phosphate pH 4.8 at 37°C for 24 h, followed by heating at 100°C for 10 min to inactivate the enzyme. The sample was centrifuged for 30 min, and the supernatant was reduced with sodium borohydride as described (Bui *et al*., 2012). The resulting reduced muropeptides were separated by HPLC (Glauner, 1988) at conditions optimized for *Listeria* muropeptides. A Prontosil 120-3-C18 AQ reversed phase column (250 × 4.6 mm, 3 μm) from Bischoff (Leonberg, Germany) was operated at 55°C at a flow rate of 0.5 ml min^−1^ with a 135-min gradient from 10 mM sodium phosphate, pH 6.0, 0.065% NaN_3_ to 10 mM sodium phosphate, pH 6.0, 15% MeOH, 0.033% NaN_3_. Muropeptides were detected by absorbtion at 205 nm. The chromatograms were analyzed using the LAURA software version 4.1.7.70 (LabLogic Systems Ltd). The main peaks were collected and analyzed by ESI-MS/MS as described (Bui *et al*., 2009). MS data obtained were consistent with previously published muropeptide structures (Boneca *et al*., 2007).

### *In vitro* virulence assays

To record invasion of *L. monocytogenes* strains into HeLa cells, an infection protocol, which we had described elsewhere, was used (Halbedel *et al*., 2012). Briefly, 10^5^ HeLa cells were seeded into the wells of a 24–multi-well plate and infected with 2 × 10^6^ bacterial cells taken from logarithmically growing cultures, which were cultivated in BHI broth (supplemented with 1 mM IPTG where necessary) at 37°C. Sampling was performed by lysing the cells in 1 ml of ice-cold PBS containing 0.1% Triton X-100 right after infection. In order to determine the number of recovered bacteria, serial dilutions were plated on BHI agar plates (containing 1 mM IPTG where necessary) and incubated over night at 37°C.

Plaque formation assays to investigate cell-to-cell spread of *L. monocytogenes* strains and assays monitoring intracellular multiplication in HeLa cells and J774.A1 mouse ascites macrophages were essentially performed as described earlier (Halbedel *et al*., 2012; 2014).

### Microscopy

Sample aliquots (0.4 μl) from logarithmically growing cultures were spotted onto microscope slides coated with a thin film of agarose (1.5% in distilled water), air-dried and covered with a coverslip. For staining of membranes, 1 μl of nile red (100 μg ml^−1^ in dimethyl sulfoxide [DMSO]) was added to 100 μl of culture, and the mixture was shaken for 20 min before the cells were prepared for microscopy. Images were taken with a Nikon Eclipse Ti microscope coupled to a Nikon DS-MBWc CCD camera and processed using the NIS elements AR software package (Nikon).

### SEM

*L. monocytogenes* strains maintained in BHI broth were sedimented at 2000 g for 2 min. The pellets were collected in 1 ml of fixative (2 h at room temperature in 1% paraformaldehyde, 2.5% glutardialdehyde in 0.05 M HEPES buffer, pH 7.2) and stored in a refrigerator at 4°C until further processing. Suspensions were sedimented at 2000 g for 10 min and washed in double distilled water. The pellets were resuspended in 100 μl of water, and 30 μl of the suspension was dropped onto a coverslip that had been covered by a carbon layer and treated with alcian blue (1% in 1% acetic acid, Sigma Aldrich) to improve adhesion. After 30 min of adsorption in a wet chamber, the coverslips were transferred to multi-well plates (12-well, TPP, Switzerland) and covered with 2.5% glutardialdehyde (TAAB, Laboratories Equipment Ltd., UK) in HEPES buffer for further fixation (30 min at room temperature). The coverslips were washed five times in water, dehydrated in an ethanol series (30, 50, 70, 90 and 96% for 15 min each and absolute ethanol for 30 min) and critical point dried (CPD 030, Bal-Tec, Liechtenstein) using liquid carbon dioxide. Finally, the sample surface was coated with a gold/palladium layer (approx. 2 nm) in a sputter coater (E5100, Polaron, UK) and examined under high vacuum conditions in a field emission SEM (Leo 1530 Gemini, Carl Zeiss Microscopy, Germany) at 5 KV using the in-lens secondary electron detector.

### Ultrathin section transmission electron microscopy

*L. monocytogenes* cells were fixed, sedimented and washed (0.05 M HEPES buffer, pH 7.2) as described above. Washed pellets were embedded in low melting point agarose (3%, mixed 1:1 with the sample; Sigma, Germany), trimmed into small pieces (about 1 × 1 mm) and fixed in 2.5% glutardialdehyde in 0.05 M HEPES buffer, pH 7.2. Samples were postfixed in osmium tetroxide (1% in water) for 60 min and 2 h in uranyl acetate (2% in water) at room temperature and dehydrated in an ethanol series (30, 50, 70, 90, 96% and absolute ethanol for 20 min). Samples were infiltrated with a mixture (1:1) of ethanol and resin (LR White hard grade; Science Services, Germany), embedded in pure resin and finally polymerized at 60°C overnight. Ultrathin sections of about 60–70 nm were cut with an ultramicrotome (Leica Microsystems, Germany), poststained with uranyl acetate followed by lead citrate. Sections were examined using a JEM-2100 transmission electron microscope (Jeol, Japan) at 200 KV. Images were recorded at full resolution of 2k × 2k with a Veleta side-mounted CCD camera (Olympus SIS, Germany).
