# Supplementary material for: Discrete and overlapping functions of peptidoglycan synthases in growth, cell division and virulence of Listeria monocytogenes
Source: Mol Microbiol. 2014 Dec 19;95(2):332–51. doi: 10.1111/mmi.12873 (PMC4320753; doi:10.1111/mmi.12873)
Supplement: Supplementary file 1 [file mmi0095-0332-sd1.pdf]

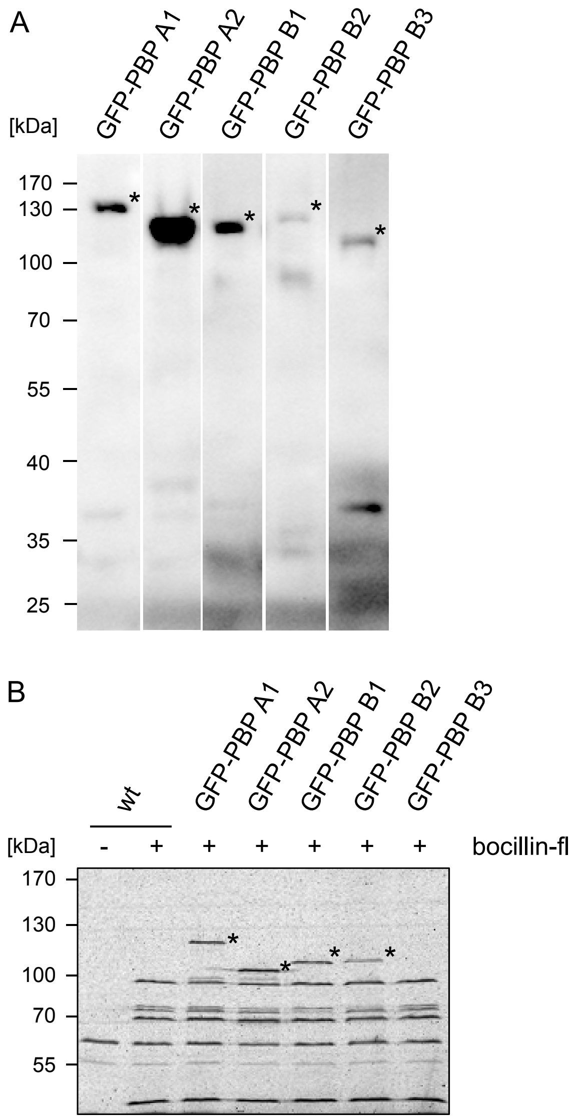

Fig. S1: Expression of GFP-PBP fusions in *L. monocytogenes* strains.
(A) Western blot to show full-length expression of GFP-PBP fusions. *L. monocytogenes* strains LMS44 (GFP-PBP A1), LMS38 (GFP-PBP A2), LMS46 (GFP-PBP B1), LMS45 (GFP-PBP B2), and LMS47 (GFP-PBP B3) were grown in BHI broth at 30°C up to mid-logarithmic growth phase. Total cellular protein extracts were separated by standard SDS-PAGE and the GFP-PBP fusions were detected in a Western blot using an anti-GFP antiserum. (B) Bocillin-fl stained SDS-PAGE gel to demonstrate penicillin binding to GFP-PBP fusion proteins. Membrane protein extracts of the same set of strains as in panel A were used. Strain EGD-e (wt) was included as control. Please note that alterations in migration patterns are explained by a reduced acrylamide concentration used for preparation of SDS-PAGE gels for PBP detection by bocillin-fl (see experimental procedures for details).

Rismondo *et al.*, Fig. S1

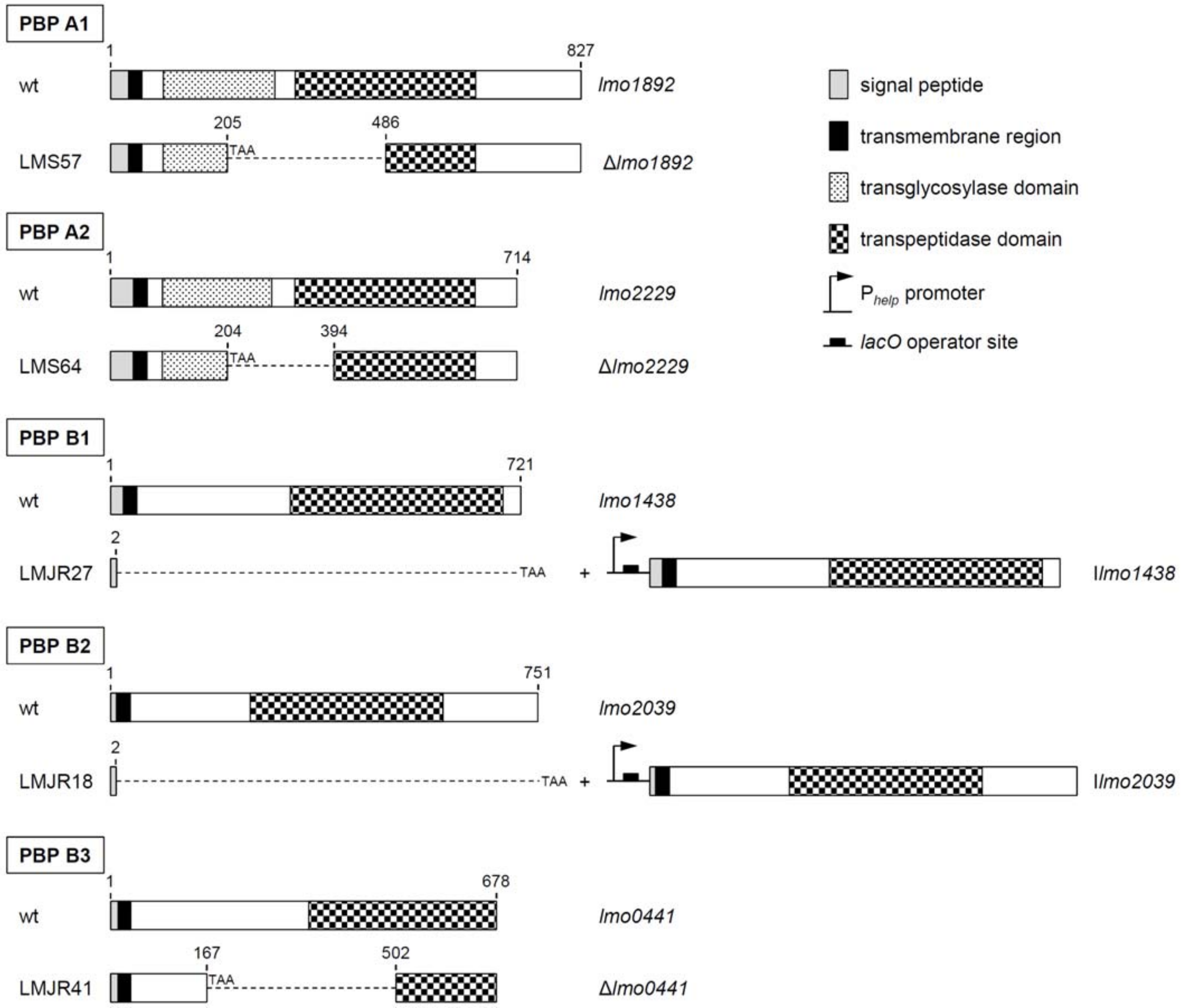

Fig. S2: Deletion of HMW PBP genes in *L. monocytogenes*.
Schematic illustration of gene regions deleted in the PBP deletion mutants, which were generated in the course of this work. Gene and strain designations are indicated on the left and genotypic descriptions on the right hand side of each illustration. Numbers above the genes indicate amino acid positions. Please note that genes encoding PBP A1, PBP A2 and PBP B3 were inactivated by deletion of internal gene regions and introduction of premature TAA codons. In contrast, the entire open reading frames were removed for genes encoding PBP B1 and PBP B2. In these strains, ectopic IPTG-inducible copies of the respective genes were present at the *attB* $tRNA^{Arg}$ sites. IPTG-dependent PBP B1 and PBP B2 mutant strains are designated I*lmo1438* or I*lmo2039* (I – inducible) throughout this study.

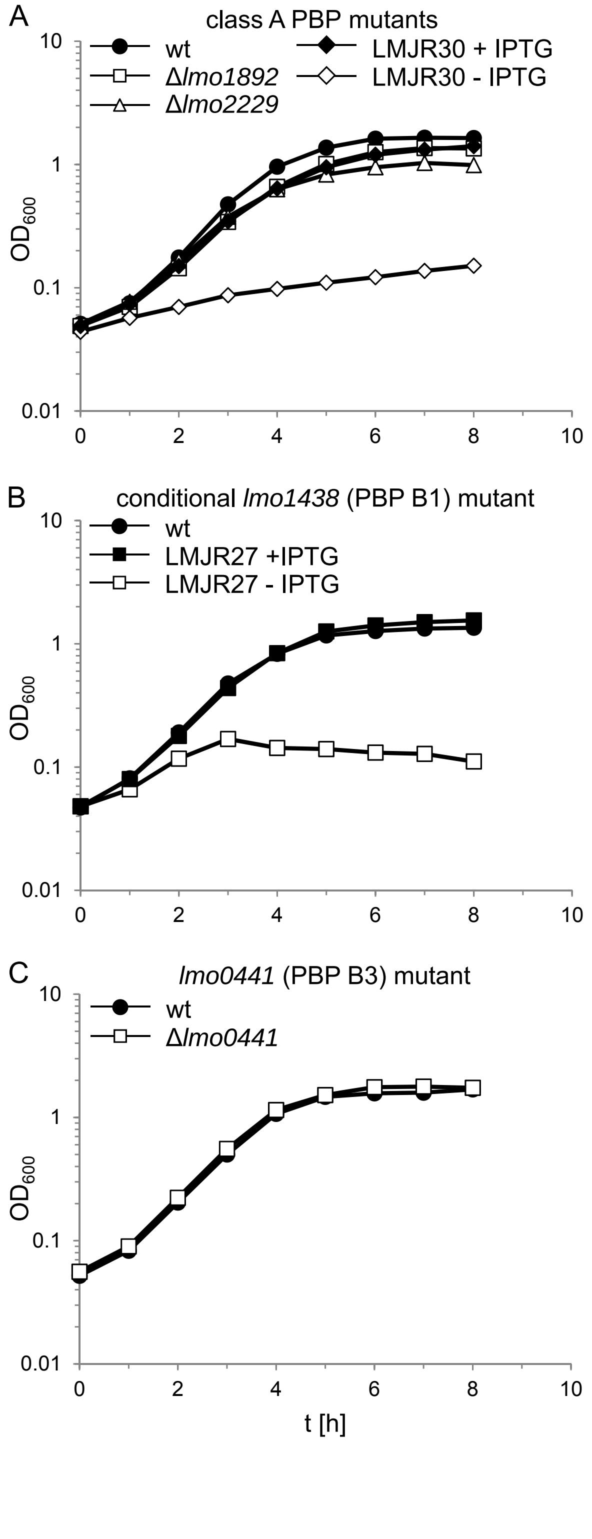

Fig. S3: Growth of *L. monocytogenes pbp* mutants at 42°C.

(A) Growth of strains lacking the class A high molecular weight penicillin binding proteins PBP A1, PBP A2 or both. Strains LMS57 (Δ*lmo1892*), LMS64 (Δ*lmo2229*) and LMJR30 (I*lmo1892* Δ*lmo2229*) where cultivated in BHI broth (containing IPTG where indicated) at 42°C and growth was recorded in hourly intervals. (B) Effect of PBP B1 depletion on heat sensitivity of *L. monocytogenes*. Strain LMJR27 (I*lmo1438*) was cultivated in BHI broth (containing 1 mM IPTG where indicated) at 42°C and growth was followed by measurements of optical density. (C) Effect of PBP B3 on growth of *L. monoctyogenes* at increased temperature as determined by growth curve analysis of strain LMJR41 (Δ*lmo0441*). Strain EGD-e (wild type) was included as a reference in all experiments.

Rismondo *et al.*, Fig. S3

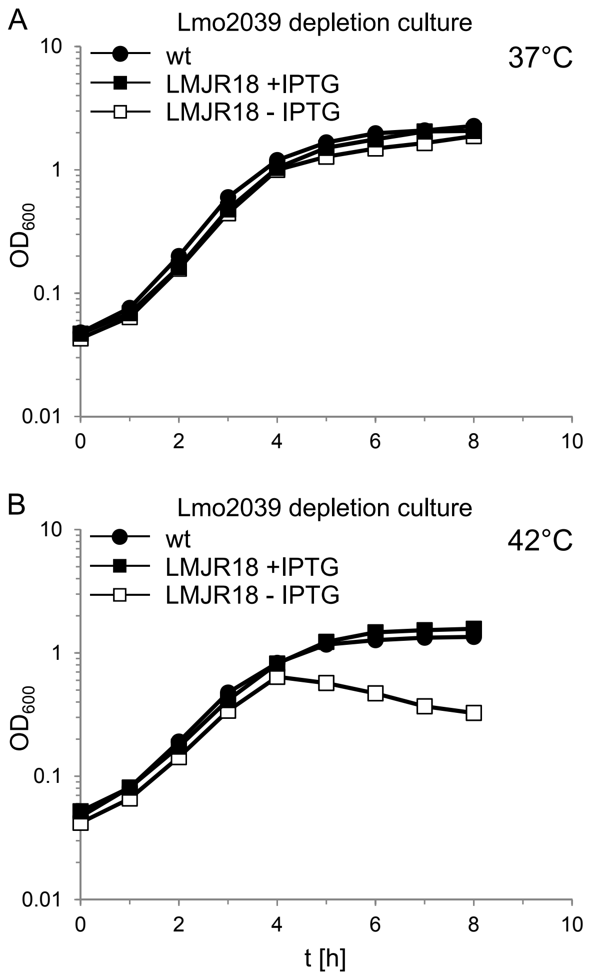

Fig. S4: Effect of PBP B2 (Lmo0239) depletion on heat sensitivity of *L. monocytogenes*.
Strain LMJR18 (I*lmo2039*) was grown over night in BHI broth containing 1 mM IPTG, washed and used to inoculate cultures containing or not containing 1 mM IPTG. Typically, no effect on growth becomes apparent at this stage of depletion when the cells are cultivated in BHI broth without IPTG at 37°C (A). However, growth of the PBP B2 depletion strain LMJR18 stopped after three to four generations during cultivation in plain BHI broth at 42°C, indicating an increased temperature sensitivity of strain LMJR18 at early stages of PBP B2 depletion.

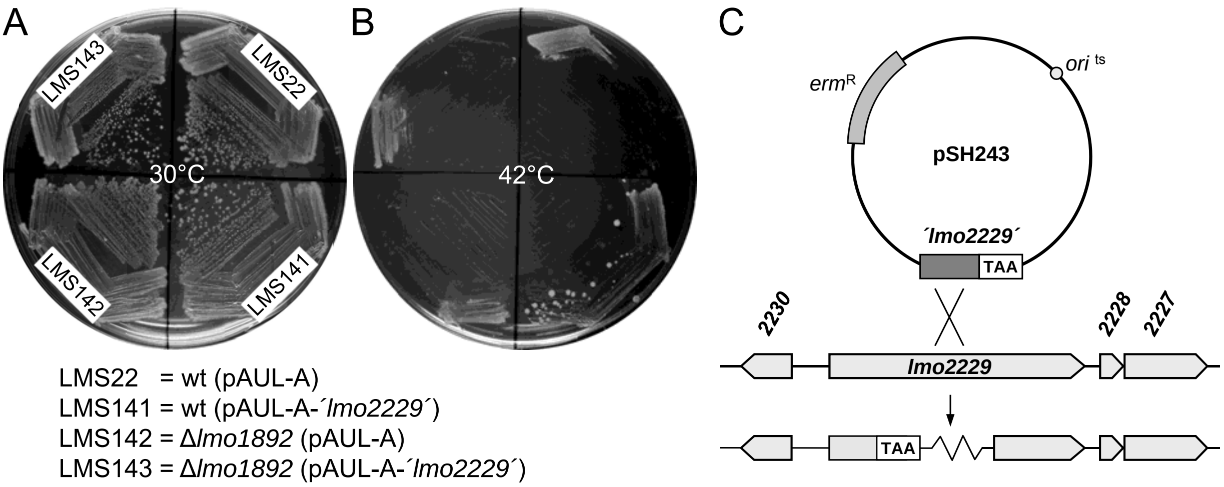

LMS22 = wt (pAUL-A)
LMS141 = wt (pAUL-A-*´lmo2229´*)
LMS142 = Δ*lmo1892* (pAUL-A)
LMS143 = Δ*lmo1892* (pAUL-A-*´lmo2229´*)

Fig. S5: Simultaneous inactivation of PBP A1 and PBP A2 is a lethal event in *L. monocytogenes*.
(A-B) Wild type and Δ*lmo1892* mutant strains either carrying the empty temperature-sensitive pAUL-A vector or the pAUL-A derivative pSH243, which was designed to inactivate the *lmo2229* gene by insertional disruption, were streaked to single colonies on BHI plates containing erythromycin and incubated at 30°C (permissive temperature, panel A) or 42°C (restrictive temperature, panel B). Colonies were only formed by strain LMS141 (*´lmo2229´* erm) but not by strain LMS143 (Δ*lmo1892 ´lmo2229´* erm) under this condition. (C) Scheme illustrating the way of insertional disruption of *lmo2229* by plasmid pSH243.

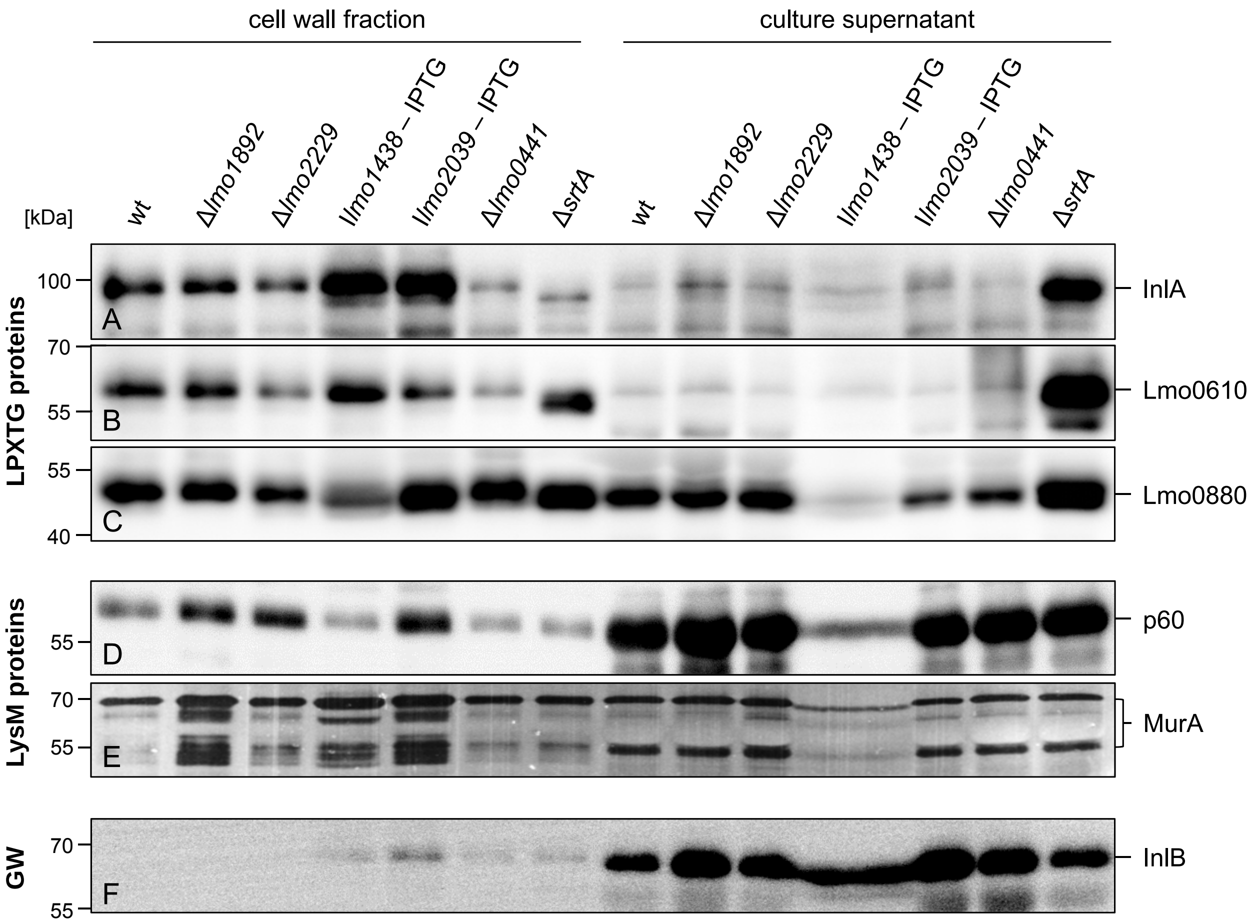

Fig. S6: Retention of surface proteins on the cell wall of *L. monocytogenes pbp* mutant strains.

L. monocytogenes strains EGD-e (wt), LMS57 (Δ*lmo1892*), LMS64 (Δ*lmo2229*), LMJR27 (I*lmo1438*), LMJR18 (I*lmo2039*) and LMJR41 (Δ*lmo0441*) were grown in BHI broth at 37°C up to an OD_{600} of 2.0 and processed as described in experimental procedures. Strain BUG1777 (Δ*srtA*) was used as control. Proteins were detected in cell wall and secretome fractions by western blotting (InlA, panel A; Lmo0160, B; Lmo0880, C; p60, D; InlB, F) or zymography (MurA, E).
